# Multi-marker metabarcoding approach to study mesozooplankton at basin scale

**DOI:** 10.1038/s41598-018-30157-7

**Published:** 2018-08-14

**Authors:** Sergio Stefanni, David Stanković, Diego Borme, Alessandra de Olazabal, Tea Juretić, Alberto Pallavicini, Valentina Tirelli

**Affiliations:** 1 Stazione Zoologica Anton Dohrn, Villa Comunale, Naples, Italy; 2 Department of Life Sciences, University of Trieste, Via Licio Giorgieri 5, Trieste, Italy; 3 National Institute of Biology, Marine Biology Station, Fornače 41, Piran, Slovenia; 4 Istituto Nazionale di Oceanografia e di Geofisica Sperimentale-OGS, Via A. Piccard 54, Trieste, Italy; 5 Institute of Oceanography and Fisheries, Šetalište I. Meštrovića 63, Split, Croatia

## Abstract

Zooplankton plays a pivotal role in marine ecosystems and the characterisation of its biodiversity still represents a challenge for marine ecologists. In this study, mesozooplankton composition from 46 samples collected in summer along the western Adriatic Sea, was retrieved by DNA metabarcoding analysis. For the first time, the highly variable fragments of the mtDNA COI and the V9 region of 18S rRNA genes were used in a combined matrix to compile an inventory of mesozooplankton at basin scale. The number of sequences retrieved after quality filtering were 824,148 and 223,273 for COI and 18S (V9), respectively. The taxonomical assignment against reference sequences, using 95% (for COI) and 97% (for 18S) similarity thresholds, recovered 234 taxa. NMDS plots and cluster analysis divided coastal from offshore samples and the most representative species of these clusters were distributed according to the dominant surface current pattern of the Adriatic for the summer period. For selected sampling sites, mesozooplankton species were also identified under a stereo microscope providing insights on the strength and weakness of the two approaches. In addition, DNA metabarcoding was shown to be helpful for the monitoring of non-indigenous marine metazoans and spawning areas of commercial fish species. We defined pros and cons of applying this approach at basin scale and the benefits of combining the datasets from two genetic markers.

## Introduction

The complexity of taxonomic composition, morphology, size range, life cycle and trophic role of zooplankton are probably unique in the marine world. Zooplankton is very important in the food webs of both marine- and fresh- water ecosystems, supporting fisheries and mediating fluxes of nutrients and chemical elements. The availability of zooplankton, food for fish larvae, is believed to be an essential factor in determining the success of fish recruitment. Any event, a decline in the zooplankton population may have far-reaching effects on the ecosystem and the economy^1^. Moreover, zooplankton can contribute to the role of marine systems as sources or sinks of CO_2_ and other greenhouse gasses^2^. Zooplankton communities are highly diverse and vary in their susceptibility to environmental stressors, such as exposure to toxic chemicals, acidification, eutrophication and oxygen depletion, or changes in temperature. As a result, the knowledge of species assemblages of the zooplankton is crucial for providing insights on the status of marine ecosystems. However, deriving qualitative and quantitative information on zooplankton composition requires intensive and highly trained labour. Classical microscopy methods are time consuming and require a high degree of taxonomic expertise and, in several cases, species level identification cannot be achieved, especially where early life stages are difficult to connect with adult forms. During the last few years, DNA barcodes provided a support to taxonomists that traditionally rely on a complex array of morphological characters to describe and discriminate species^3^. In this context, the growing database of DNA barcodes linked to species names and morphological characters for marine zooplankton may be considered as a “Rosetta Stone” for decoding patterns of species diversity in the pelagic realm^4^). DNA barcodes are also useful to discover new species, reveal cryptic species, and assess taxonomically significant variation within species with broad or disjoined distributions^5^.

The introduction of high–throughput sequencing (HTS) technology based on loop array sequencing allows for analysis of a large number of samples simultaneously. Its large scale sequencing capacity and low costs holds considerable promise for diversity and biodiversity monitoring in the context of global change^6^. This technology has been applied successfully to several studies on marine plankton communities^7–16^ initially using 454 FLX Titanium pyrosequencer (Roche), followed by Illumina technology.

DNA metabarcoding relies on the completeness and quality of the reference database used for taxonomic assignments, which should ideally provide a complete representation of all taxa from a given habitat^17^. However, this is overambitious and not feasible when it comes to zooplankton as a huge number of different species, belonging to various different phylogenetic groups, characterizes this biome. Even when using GenBank, which is the most redundant sequence repository, the coverage of target species is not satisfactory, as the available reference sequences cover only a fraction of marine zooplankton^5,11^. Together with the improvement of indigenous barcode databases^18^, the comparison and combination of different barcode markers could contribute to a more effective taxonomic assignment of DNA metabarcoding of zooplankton.

The mtDNA COI molecular marker is considered the universal animal barcode^19^ while the 18S marker is often a standard when considering marine microbial eukaryotic diversity^20^. Since many zooplankton species have reference sequences for only one or the other of these two markers deposited in the GenBank, the single reference database are limited and could be improved by databases combination. Recent studies report the use of both DNA markers to assess marine^21^ and zooplankton diversity^22,23^. In this study, we combined the taxonomy assignment of the two DNA metabarcoding markers in a single abundance matrix to efficiently compile an inventory of mesozooplankton diversity. Reads were obtained using a Personal Genome Machine (PGM) Ion Torrent (Life Technologies). This approach was tested, for the first time, on a large dataset consisting in 46 mesozooplankton samples collected during summer 2014 in the Italian territorial waters of the Adriatic basin, from the Gulf of Trieste to the Gargano Peninsula. Results from this study show that this multi-gene metabarcoding approach is very effective for large-scale biodiversity assessments as confirmed by the very high number of matches obtained, if compared to the use of a single marker. Additionally we assessed the reliability of this methodology in comparison with classical morphological identification of specimens. Moreover, the efficiency of the DNA metabarcoding approach as a monitoring tool for commercial species as well as non-indigenous (NIS) marine metazoans, currently present or deemed likely to invade the Adriatic Sea (early warning detection), was evaluated.

## Results

### Molecular sequence datasets, taxonomical assignment and molecular operational taxonomic units

From the 46 samples analysed, one sample (16) for COI and three samples (43, 46 and 52) for 18S did not produce any amplification although several attempts were made, including different trials of genomic DNA extractions. Therefore, these samples were not included into the combined (COI + 18S) analysis of mesozooplankton diversity. Overall diversity estimates obtained by each separate marker resulted higher for COI compared to those obtained with 18S (Table S1), mostly due to a better-suited reference database for COI. The proportion of sequences that did not find a hit with the reference sequences available in GenBank also differed substantially between the two genes targeted (58.4 and 61.3% for COI and 22.4 and 28.0% for 18S, at 90 and 95% similarity thresholds, respectively).

Sequencing effort was greater in COI (1,198,723 raw sequences) than in 18S (539,297 raw sequences) with numbers reduced to 824,148 (COI) and 223,273 (18S) after stringent quality filtering. The number of reads differed either among samples or between the two marker genes (Fig. S1). The average number of sequences were 17,916 for COI (min: 7,033 in sampling site 6 and max: 38,198 in site 26) and 5,192 for 18S (min: 528 in sample 6 and max: 10,710 in site 66).

The taxonomical assignment of reads against an in-house reference sequence database (388,804 and 65,232 sequences for COI and 18S, respectively), conducted at various similarity thresholds, recovered in total 419 taxa (72 of which were identified by a single read) (Table S1). The combined dataset, built using the similarity thresholds of 95% for COI and 97% for 18S and manually polished for misidentifications, comprised of 234 taxa.

In terms of performance of the two markers, and in order to identify the barcoding gap for the two genes, the number of assigned reads steeply increased from 100 to 95% (COI) and to 97% (18S) reaching the first plateau followed by a new appreciable increase between 88% and 85% (for COI) and 87% and 86% (for 18S) (Fig. S2). No further reads were assigned between 69 and 70% (in COI) and 80 and 79% (in 18S). The cut-off value (the similarity thresholds at which none of the assigned reads still did not matched more than one Phyla) was 80% for the COI and 86% for the 18S reads. Based on the COI sequencing, the mesozooplankton community was composed by Arthropoda (40.8%), Cnidaria (29.0%), Chaetognatha (7.6%), Echinodermata (5.7%), Chordata (4.4%), Mollusca (3.6%), Annelida (3.5%), other Phyla (0.3%) and non-metazoan (5.1%). For 18S the proportions were: Arthropoda (38.5%), Cnidaria (22.9%), Chordata (21.8%), Chaetognatha (3.4%), Echinodermata (2.2%), Annelida (1.4%), Mollusca (0.6%), other Phyla (1.3%) and non-metazoan (7.9%, Fig. S2).

Taxonomy free clustering into molecular operational taxonomy units (MOTUs^24^) recovered 23964, 28591 and 34096 MOTUs using 90, 95 and 97% dissimilarity levels for the COI reads and 1150, 1448, 1714 and 2554 MOTUs using 90, 95, 97 and 99% dissimilarity levels for the 18S reads. However, when using “only metazoa” reads (discarding sequences that did not produce hits to any metazoan reference sequence at 80% similarity level for COI and 86% for 18S) the numbers of MOTUs recovered were considerably lower and more comparable between both markers. We obtained 1259, 2020 and 3793 MOTUs using 90, 95 and 97% dissimilarity levels; and 570, 759, 928 and 1580 MOTUs using 90, 95, 97 and 99% dissimilarity levels for COI and 18S, respectively. All the MOTU clusterings included a chimera checking^25^ and LULU curation step^26^.

### Spatial variability of Adriatic mesozooplankton community

**Figure 1:**
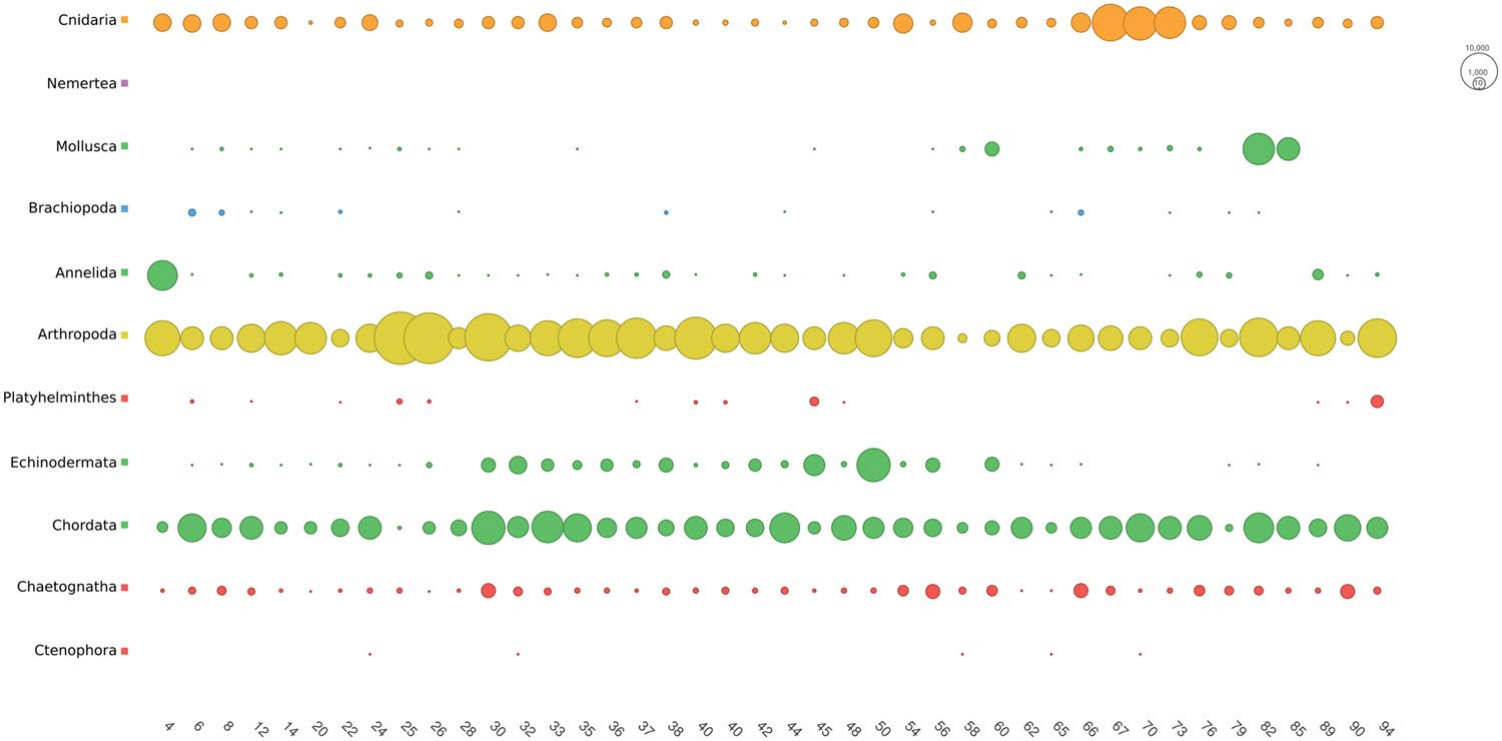
Bubble plot showing the distribution of the reads of the combined dataset (COI + 18S) for each phylum and among samples.

The combined dataset indicated that in late summer the mesozooplankton community was dominated by Arthropoda followed by Chordata, Echinodermata and Cnidaria (Fig. 1).

**Figure 2:**
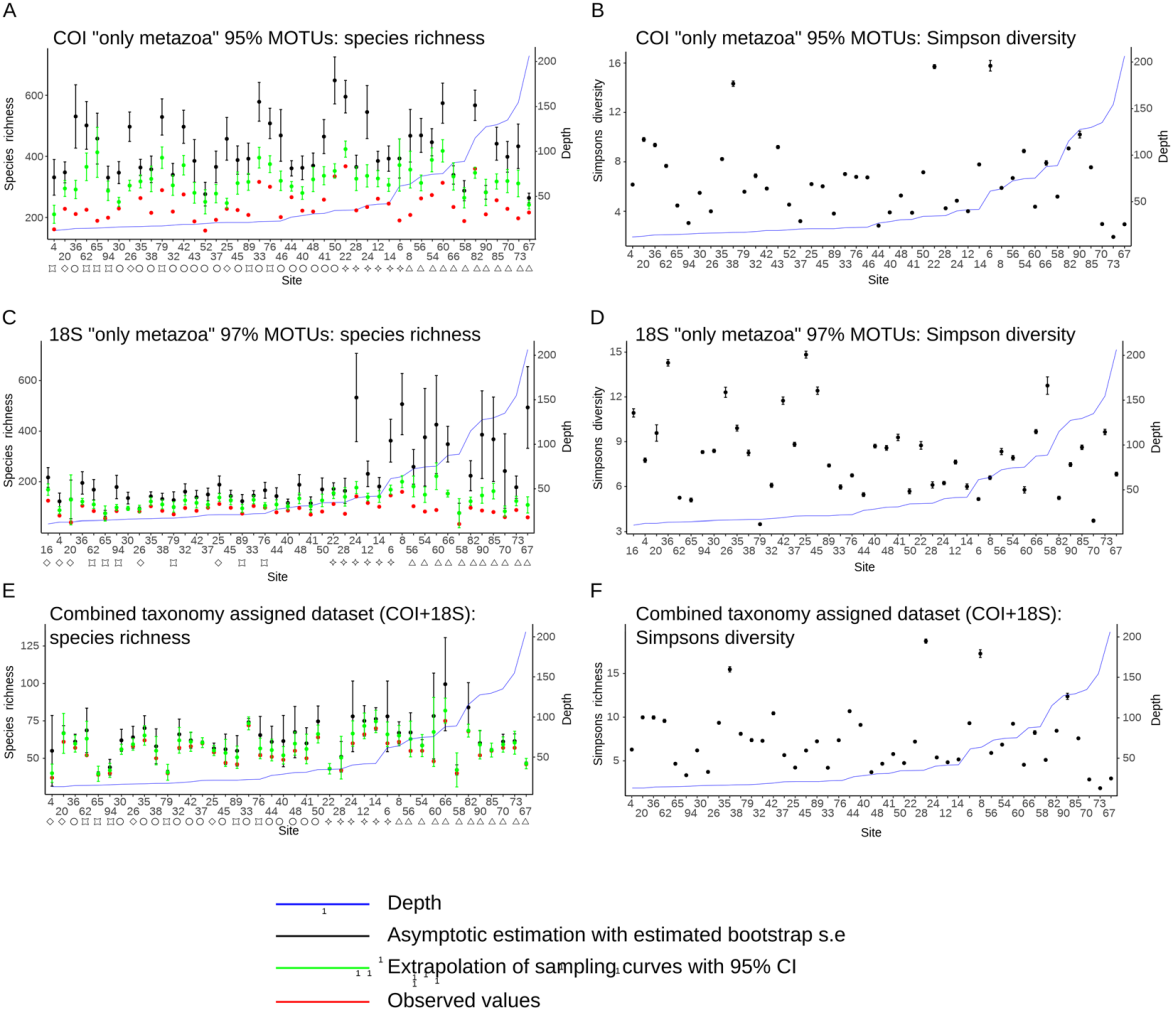
Alpha diversity size-based estimation from species richness (**A**,**C**,**E**) and Gini–Simpson index (**B**,**D**,**F**) according to: COI “only metazoa” dataset clustered at 95% similarity level (**A**,**B**) 18S “only metazoa” dataset clustered at 97% (**C**,**D**), and the combined taxonomically assigned dataset (COI + 18S; **E**,**F**). Species richness was estimated asymptotically, by extrapolation to the highest number of reads per site and from the observed values, while Simpson’s diversity was estimated only from asymptotes. Error bars report 95% confidence intervals for extrapolated values. Standard error for asymptotic estimations were estimated by bootstrapping.

Asymptomatic estimation of species richness calculated from 18S datasets revealed significantly higher diversity values in sampling sites from deeper waters. However, such differences in value between shallow and deep water sites are less clear when looking at the asymptotic estimations of other datasets (COI and combined – COI + 18S) and they disappear when estimating the diversity with sampling curves extrapolated to the highest number of reads per site (Figs 2, S7, S9). No apparent pattern is visible exploring the alpha diversity plot from the Simpson-Gini index (Figs 2, S8, S10), however, for the 18S datasets, it appears that in shallow water sampling sites there is larger variability compared to values from sites in deeper waters.

**Figure 3:**
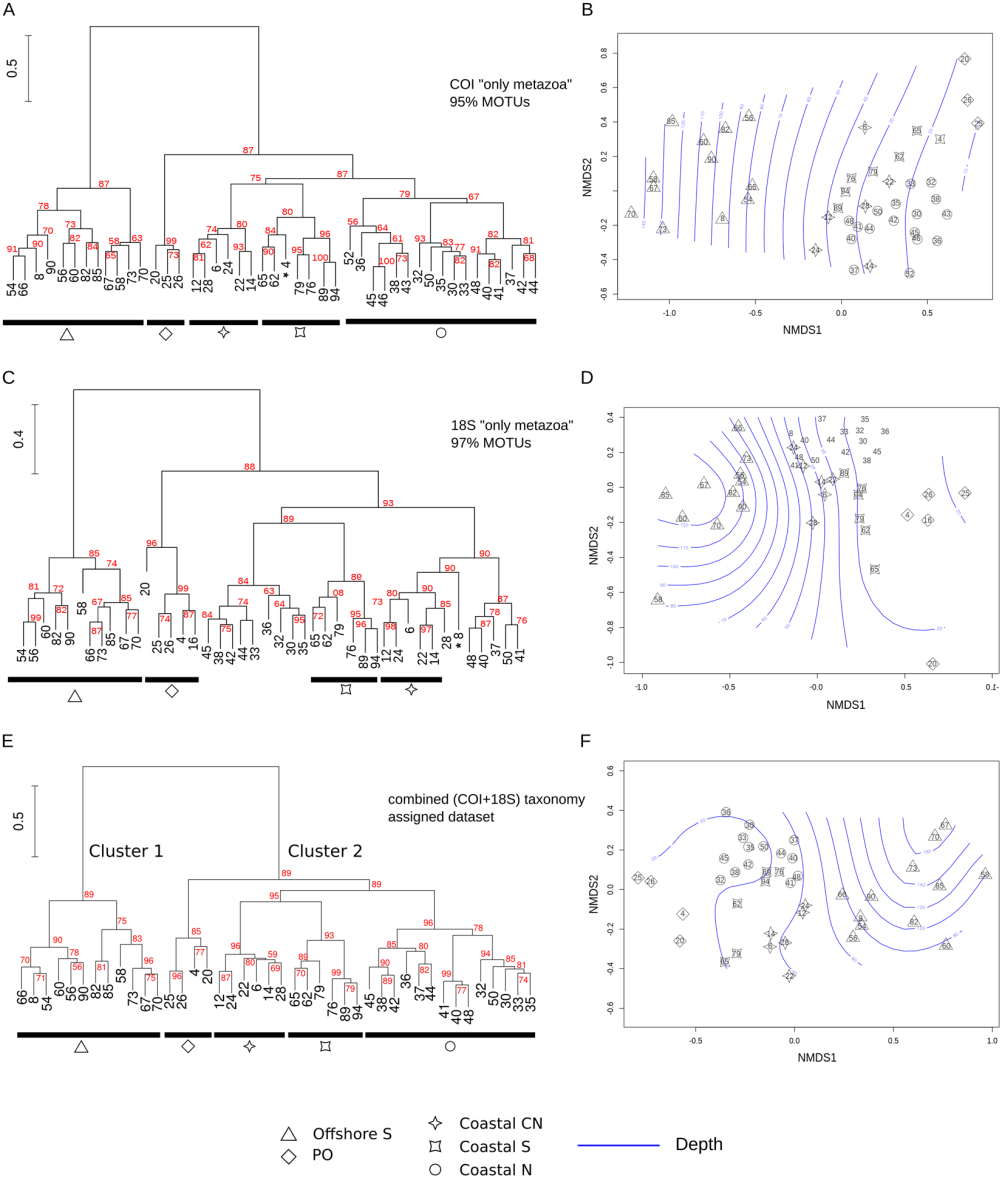
Beta diversity estimated by clustering analysis (**A**,**C**,**E**) and NMDS plots (**B**,**D**,**F**) according to COI “only metazoa” dataset clustered at 95% similarity level (**A**,**B**), 18S “only metazoa” dataset clustered at 97% (**C**,**D**), and the combined taxonomically assigned dataset (COI + 18S; **E**,**F**). Stability of each node was evaluated values were estimated by an Approximately Unbiased test (values in red). Bathymetry is superimposed (blue contour lines) on the NMDS plots according to the depth measurements at the sampling sites (Table S3).

**Figure 4:**
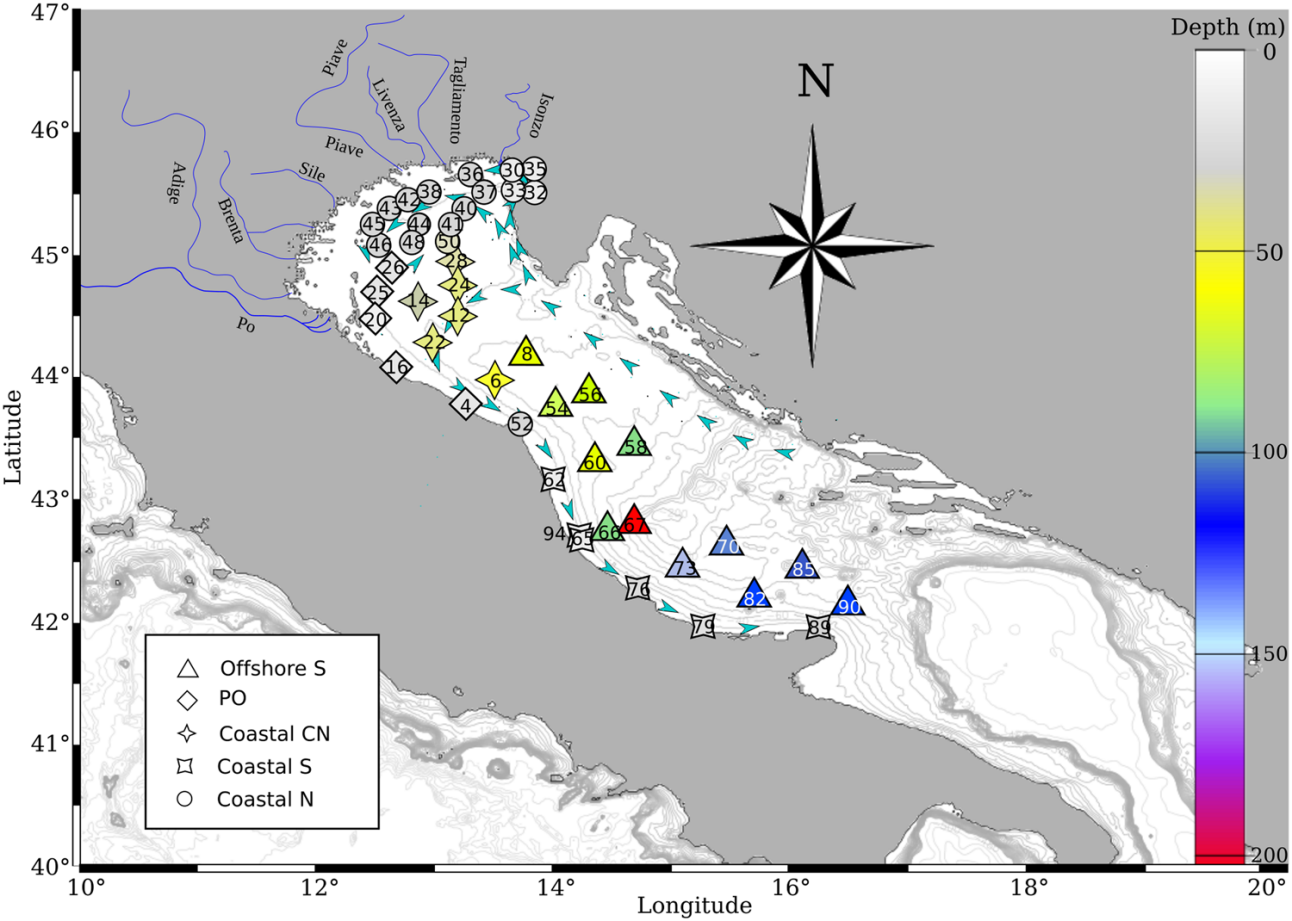
Map indicating the location of the sampling stations, colour coded accordingly to bottom depth. More details on sampling dates, time, locations and depths is given in Table S3. Map created using R^50^ package marmap 0.9.6^58^ querying for the ETOPO1^59^ bathymetric dataset hosted on the NOAA server (National Oceanic and Atmospheric Administration, http://www.noaa.gov). Symbols at sampling sites match those from clustering analysis (Fig. 3), while their colour matches the depth chart.

The beta diversity visualised with non-metric multidimensional scaling plots (NMDS; Figs 3, S3, S4) and with Ward’s hierarchical clustering (Figs S5, S6) produced a similar pattern in all of the MOTU datasets no matter the DNA marker, similarity levels used for clustering, nor the taxonomic filtering (“only metazoa” *vs*. “no taxonomic filtering”). Furthermore, this pattern was also consistent with the observations in the taxonomically assigned combined dataset (COI + 18S; Fig. 3E,F). The sampling sites were grouped in two main clusters: one aggregating the 12 offshore sampling sites (Cluster 1, Figs 3, 4) and a second grouping 30 samples collected in shallow water (Cluster 2, Fig. 3). Cluster 2 is further separated into four subclusters. Two of these (named PO and Coastal N; Figs 3, 4), include sampling sites located in proximity of rivers’ estuaries (from west to east, Po, Adige, Piave, Isonzo, Brenta, Livenza, Tagliamento and Sile River). Samples distributed along the river Po outflow grouped together, which is also clear in the NMDS plots (Figs 3, 4). The third subcluster (Coastal S; Figs 3, 4) grouped coastal sites distributed along the more southern Italian coastline (south of Ancona) while the forth one (Coastal CN; Figs 3, 4) includes sites from the central-north Adriatic. The only substantial difference between the datasets is that, according to 18S, the stations belonging to the Subcluster Coastal N are here divided into two groups (Figs 3, S5, S6).

SIMPER test showed that dissimilarity between Cluster 1 (Offshore S) and Cluster 2 (PO, Coastal N, Coastal S and Coastal CN) (average dissimilarity = 63.49%) was mainly due to the higher abundance of the cladocerans *Penilia avirostris* and *Pleopis* sp., calanoid copepods *Temora stylifera, Paracalanus parvus, Acartia (Acartiura)* *clausi* and *Centropages* in Cluster 2. Contrary, the offshore cluster (1) was more abundant with the siphonophore *Nanomia bijuga*, the chetognath *Flaccisagitta enflata*, the tunicate *Doliolum nationalis* and the cyclopoid copepod *Oithona similis*. In Cluster 2 the difference between the Subcluster PO and the subclusters Coastal S (average dissimilarity = 56.34%), Coastal CN (average dissimilarity = 57.34) and Coastal N (average dissimilarity = 58.23%) was mainly due to the increase in abundance of the cladoceran *Pleopis* sp., the calanoid copepods *Paracalanus parvus* and *Centropages* in the Subcluster PO. The difference between the subclusters Coastal N and Coastal CS (average dissimilarity = 50.91%) and Coastal N and Coastal CN (average dissimilarity = 52.90%) was linked with the higher abundance of echinoderm larvae (*Paracentrotus lividus*, *Psammechinus miliaris* and *Ophiothrix fragilis*) and anchovy eggs and larvae in the northern samples. Finally, the difference between the subclusters Coastal CN and Coastal S was the smallest (average dissimilarity = 50.51) and was mostly linked with the higher abundance of *Temora stylifera* and *Penilia avirostris* and lower abundance of *Paracalanus parvus* in the samples from the coastal S.

**Figure 5:**
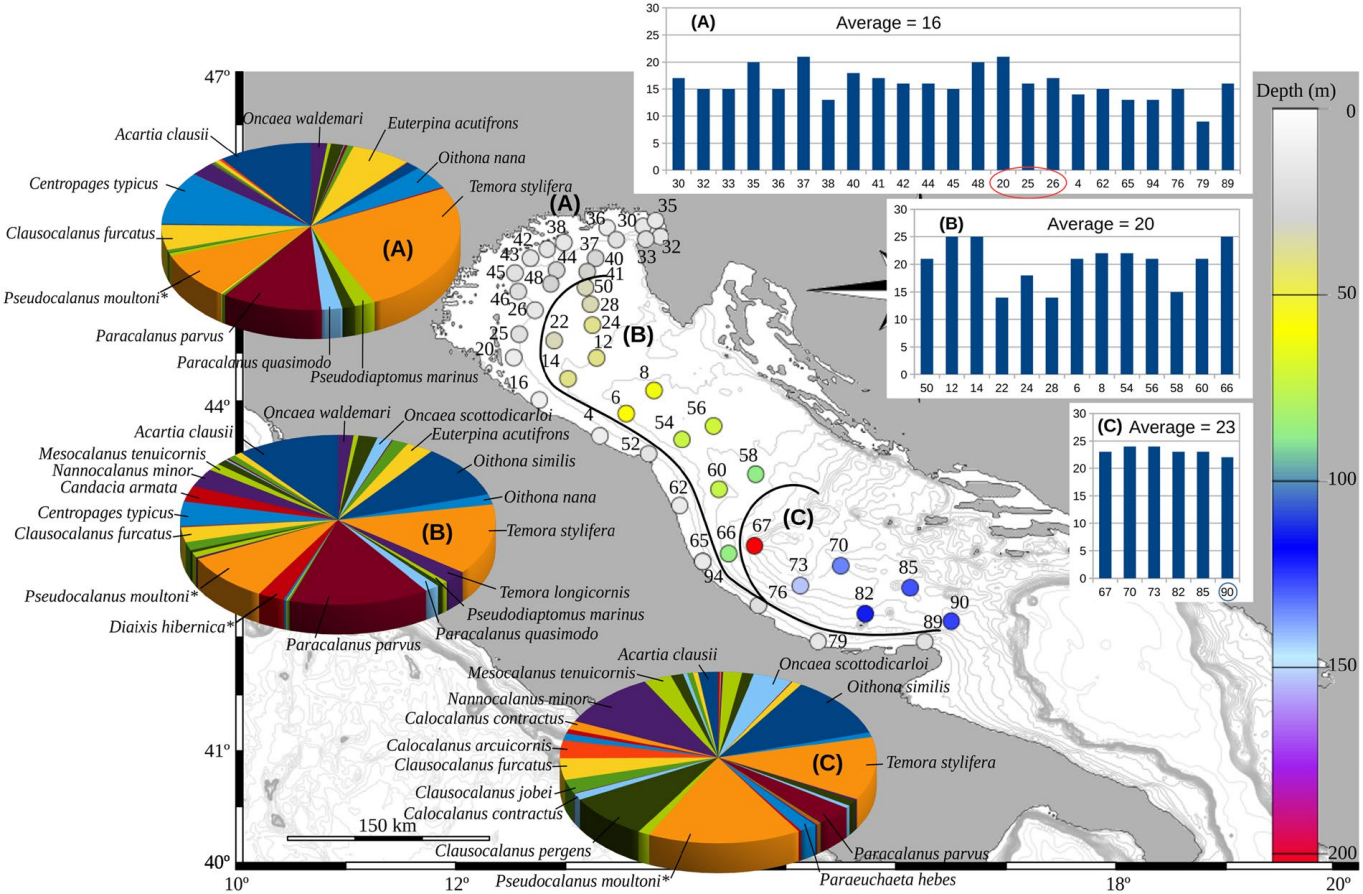
Spatial distribution of Copepods comparing coastal (**A**) to offshore northern (**B**) and southern (**C**) areas. The histograms on the right are reporting the species richness at each sampling site, while the pie charts are indicating the species composition for each of the three areas. Map created using R^50^ package marmap 0.9.6^58^ querying for the ETOPO1^59^ bathymetric dataset hosted on the NOAA server (National Oceanic and Atmospheric Administration, http://www.noaa.gov). *Indicates species that most probably were misidentified (see text and Table S1).

Spatial variability was also explored considering only Copepoda (47 taxons in total), one of the most abundant and studied group in zooplankton ecology (for the Adriatic Sea^27^). The Ward’s hierarchical clustering identified three groups of samples (Fig. 5): group A, formed by all the coastal and northernmost samples, group B including all the northern and central off-shore samples and group C identified by the southern off-shore samples. It is noticeable that, moving from north to south and from coastal to offshore, there was a gradual increase of the number of species (from 17 to 24, as average values) with the appearance of new taxa and a change in the contribution of each species to the three groups of stations (Fig. 5). This north–south and coastal–offshore trend was not visible when considering the same grouping of samples from the entire mesozooplankton dataset (data not shown). It should be noted that this analysis was made only on the molecular sequence datasets: some species were probably misidentified, as in the case of *Diaixis hibernica* (probably *D. pygmaea*) and *Pseudocalanus moultoni* (probably *P. elongatus*) (see Table S1 for details).

**Figure 6:**
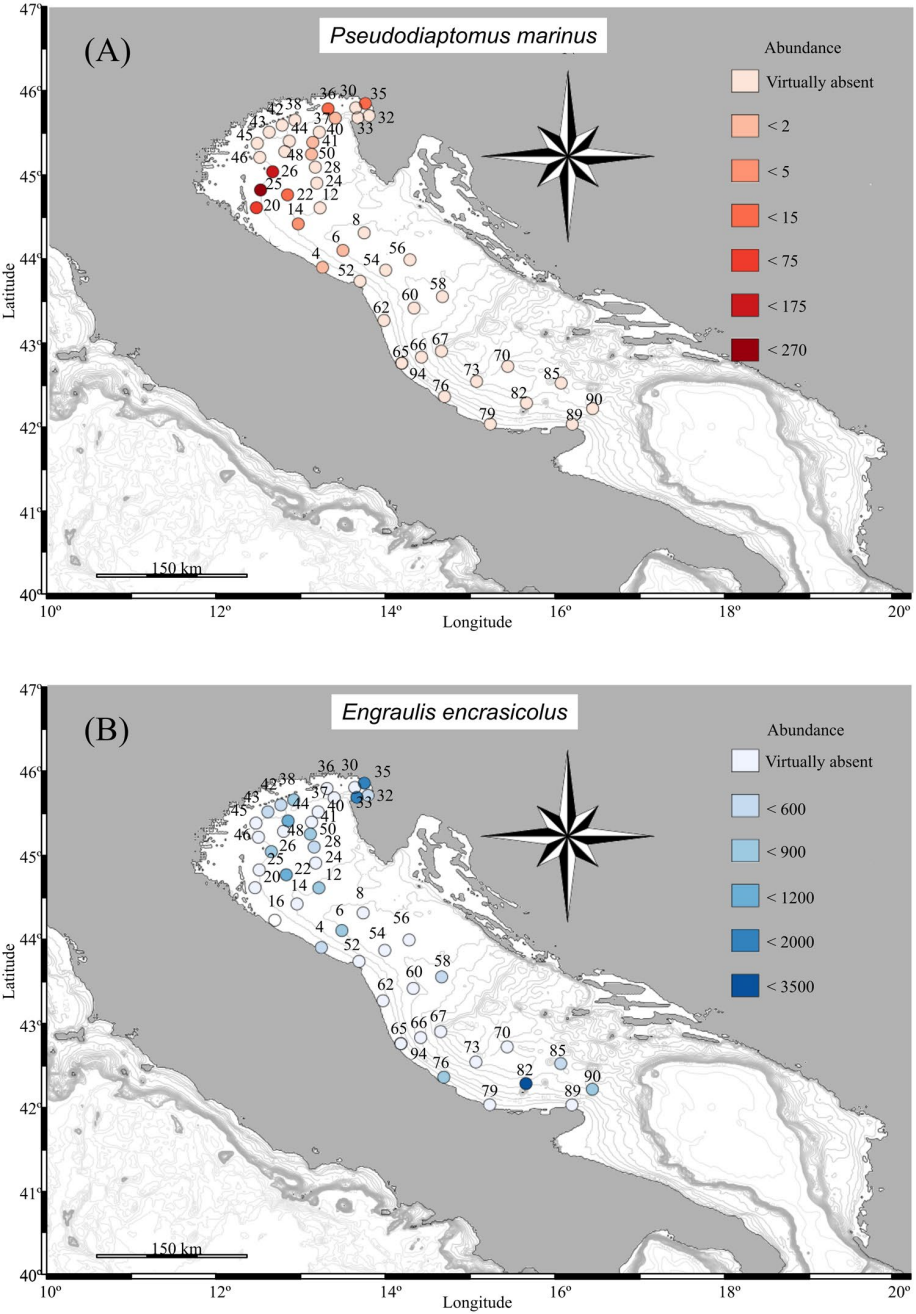
Distribution obtained by the relative abundance of sequences for: (**A**) *Pseudodiaptomus marinus* and (**B**) *Engraulis encrasicolus*. Maps created using R^50^ package marmap 0.9.6^58^ querying for the ETOPO1^59^ bathymetric dataset hosted on the NOAA server (National Oceanic and Atmospheric Administration, http://www.noaa.gov).

In this study we have cross-matched the list of species reported in Zenetos *et al*.^28^, evaluated the GenBank taxonomical assignment at progressively higher stringency minimum level of identity (90, 95 and 97%) to check the taxonomic assignments, and highlighted all the unexpected species for the Mediterranean Sea as potential invasive species (Table S1). Molecular analysis, both with COI and 18S reads detected the presence of the NIS copepod *Pseudodiaptomus marinus* in several stations located in the northern basin at a high degree of similarity (97% for COI and 99% for 18S (Fig. 6A, Table S1). Among these, the station 35 (Gulf of Trieste) was also screened by morphological analysis, confirming the presence of *P. marinus*. On the other hand, a single specimen was found at station 76 (southern Adriatic) which was negative for genetics.

In order to tackle the relative abundance of commercial important species (in terms of number of reads), the fish *Engraulis encrasicolus* was also plotted (Fig. 6B). The molecular approach revealed higher numbers of reads in already known spawning areas of this species (this survey was carried out during the reproductive period of *Engraulis encrasicolus*): the Gulf of Trieste (stations 33, 35) and several other sites in the northern and central Adriatic^29^. Of particular interest is the confirmation of a high abundance of early life stages of anchovies in the southern Adriatic, in proximity of the Tremiti islands (sample 82^29^).

### Comparison of DNA metabarcoding and morphological taxon identification

**Figure 7:**
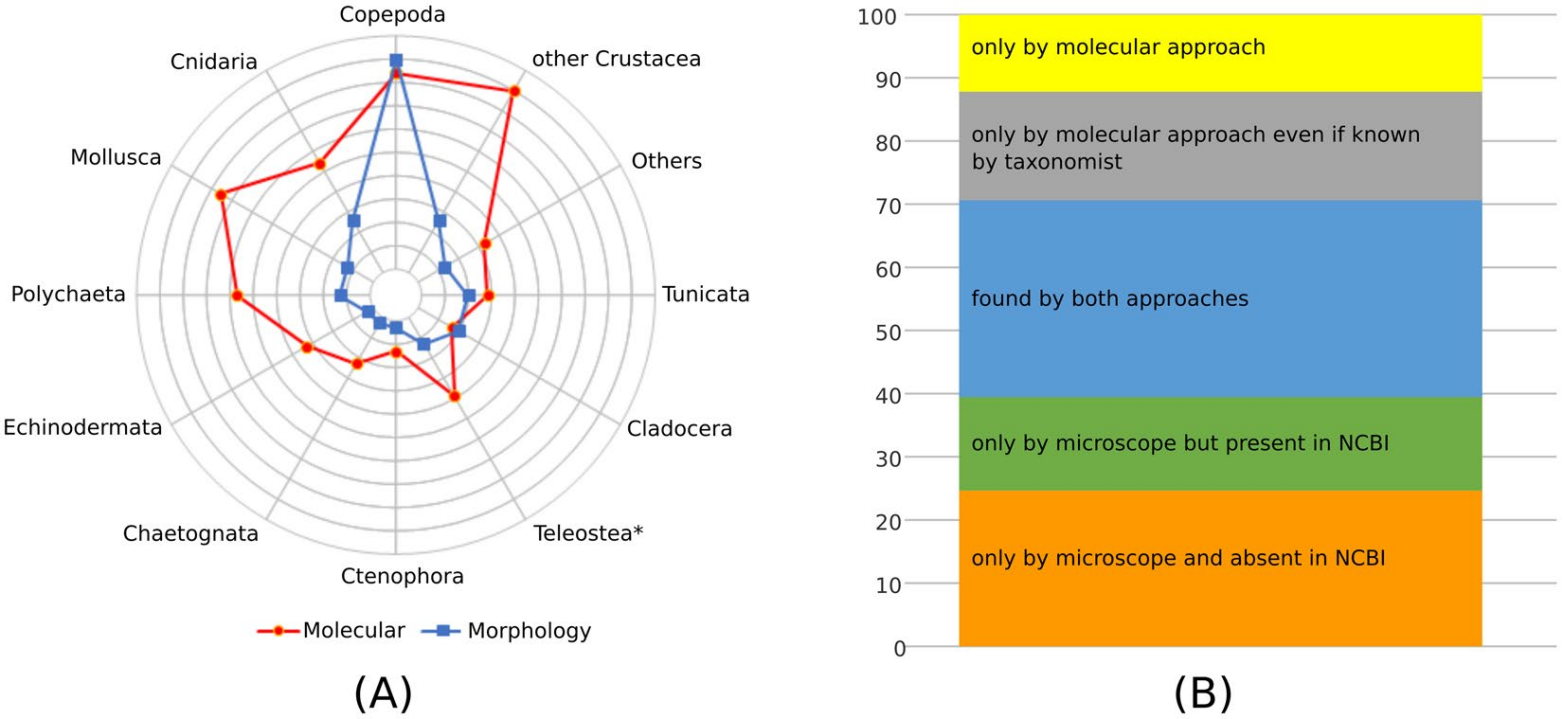
Graphical comparison on the performance of molecular and microscope analyses in retrieving taxonomical units. (**A**) Overall performance by the two approaches by major groups (* by visual inspection it is possible to distinguish 3 groups: *Engraulis encrasicolus* eggs, other fish eggs and fish larvae); (**B**) proportion of copepod species identified by the two approaches considering if the sequences were available or not in the GenBank database or expected to be present in the samples.

Detailed morphological analysis and zooplanktonic taxa identification were performed at the stereomicroscope on 8 formalin-preserved samples (stations 24, 35, 56, 60, 62, 73, 76 and 82) collected through the whole investigated area (Fig. 4). A total of 8,338 organisms were screened and 87 taxa were identified as Phyla (5 OTUs), Class (5), Infraclass (2), Order (9), Family (1), Subfamily (1), Genus (13) and Species (51). In order to evaluate the detection power of the molecular approach *vs*. the traditional morphological analysis, we organized the datasets into 12 major taxonomic groups applicable to the two approaches (Fig. 7A). The molecular approach always identified a higher number of OTUs except for the groups of cladocerans and copepods, which are commonly recognized at species level through microscopic observations. At the stereomicroscope many meroplanktonic larvae were recognised only to class (as Bivalvia) or order (as Decapoda) or phylum (as Echinodermata) level, whereas DNA metabarcoding analysis was often able to determine the species (see Table S1). For this reason, a thorough comparison between morphological and DNA metabarcoding approaches was carried out only considering the organisms belonging to Cladocera and Copepoda. Overall, from morphological analysis, cladocerans and copepods represented 44.16 ± 23.00% and 28.27 ± 1 9.33% (mean ± standard deviation) respectively of total community in terms of abundance (ind m^−3^). Merging morphological and DNA metabarcoding results, we obtained 283 identification records belonging to 67 taxa: 32% of the records were detected by both methods, 39% only by the morphological approach (including 13% of records whose sequences are present in GenBank database) and, finally, 29% only by the molecular approach (including 17% of records corresponding to taxa commonly recognized by taxonomist) (Fig. 7B). Overall, 6 species of cladocerans were identified by the observation at the stereomicroscope (Table S2) of which only *Pseudevadne tergestina* and *Evadne nordmanni* were not recognized by the molecular approach due to missing reference sequences. The list of copepods includes 57 species and 4 genera. Morphological analysis allowed identification of 54 taxa while the DNA metabarcoding revealed approach only 40, mostly retrieved by COI (Table S2). Three species found only by the DNA metabarcoding approach were pointed out as possible misidentifications (Table S1) as they were typical of non-Mediterranean habitat (*Calanus finmarchicus, Pseudocalanus moultoni* both retrieved by 18S) or present in the Adriatic as a different species not yet barcoded (*Diaixis hibernica* retrieved by COI might actually be *D. pigmaea*, which is confirmed for the Adriatic). The NIS species *Pseudodiaptomus marinus* was detected by both methodologies at station 35 (retrieved by both COI and 18S) and only by taxonomy at station 76. Although *Centropages* is often very abundant in the Adriatic, *C. kroyeri* was identified only at the stereomicroscope – the genetic approach was not able to discriminate species belonging to this genus (retrieved only with 18S). The genera *Sapphirina* and *Farranula* were found only by the morphological approach while *Microcalanus* and *Tritia* (both retrieved by COI) only by the DNA metabarcoding approach.

## Discussion

With the development of HTS platforms, we have experienced a technological revolution and several studies have applied DNA metabarcoding approaches for quantifying the relative diversity of various taxonomic groups of metazoans in comparison with traditional morphological approach (see review by Leray & Knowlton^30^). Our study demonstrated that despite various methodological biases, both molecular datasets are comparable with respect to zooplankton taxa recovered (Table S2). However, when considering taxonomy free MOTU clustering approach we recovered a considerably higher overall alpha diversity with DNA metabarcoding – 2020 and 928 MOTUs were recovered using 95% similarity threshold for COI reads and 97% for 18S, respectively. Although the values obtained for mesozooplankton richness are dependent on the choice of pruning method for reads, clustering algorithm and threshold, they can be considered high as the total number of described metazoan species for the whole Mediterranean Sea is ca. 11,000^31^. On the other hand, numbers of MOTUs recovered by our approach is considerably lower then the numbers of MOTUs recovered in a comparable study, where COI and 18S metabarcoding approach was used to investigate littoral hard-bottom communities^21^. It is hard to evaluate what the reasons are for such differences, as both studies targeted different communities (mesozooplankton vs. eukaryotic biodiversity of benthos) and used different bioinformatic pipelines and clustering algorithms. An explanation could be that our study incorporated an additional post-clustering curation step for removing erroneous MOTUs^26^ (LULU method) by which we have greatly lowered the number of overall MOTUs (from 23317 and 5760 to 2020 and 928 for COI and 18S respectively) while retaining the number of reads at the same time.

Methodological constrains of the molecular approach is mainly linked to the incompleteness of molecular reference databases and to the fact that zooplankton species often have reference sequences deposited in the GenBank for either COI or 18S marker. Furthermore, our results confirm^18,32^ that COI ensures detection of species-level diversity. However, while this marker has by far the most reference sequences available, it in many cases requires group-specific primers to ensure representative amplification through the animal tree of life^3,33^. In this study a dual COI–18S markers approach was successfully applied to a large number of mesozooplankton samples collected over the Adriatic Sea. This study also represents the first mesozooplankton survey carried out at basin scale combining molecular and morphological taxonomy. Our approach not only improved the taxonomic assignment of zooplankton, but it was effective in tackling commercial species and also NIS marine metazoans. Presented results confirmed that the efficiency of the DNA metabarcoding estimated in terms of the correct taxonomical assignments of reads is mostly affected by the paucity of the reference database, in case of closed reference OTU pickings. Exploring single marker efficiency, within the phylum Chordata, COI sequences perform better in retrieving vertebrate taxa, while 18S worked well with chaetognaths and tunicates (67.6% to Appendicularia and 30.3% to Thailacea). This discrepancy can be explained by the under representation of COI sequences deposited in GenBank database for these two taxonomical groups (Appendicularia has only 1 species vs. 7 and Thaliacea has 3 vs. 30, in COI and 18S respectively) and by the fact that many tunicates require group specific primers for amplification of COI^34^. Within all other phyla both COI and 18S did well but only a small number of species was recognised by both markers (Table S1). We tried to overcome these limitations by combining two separate DNA metabarcoding markers (COI and V9 variable region of the 18S) datasets into a single matrix.

The efficiency of such a multi-gene metabarcoding approach for large-scale biodiversity assessments is confirmed by the high number of taxonomic assignments – 234 taxa (mostly species) of mesozooplankton. Outcomes of DNA metabarcoding studies are affected not only by primers over-specificity, and paucity of reference databases, but also by the selection of similarity thresholds. While a minimum similarity threshold that is too low leads to misidentification and false positives, a threshold that is to high risks not recognizing a species even if its sequence is deposited in the reference database (due to intraspecific variability of the gene). After testing a range of similarity thresholds for taxonomy assignments (Table S1) and for the construction of the combined dataset for downstream statistical analysis, we decided to combine the 95% similarity threshold for the COI and 97% for the 18S. Although these selected thresholds are comparable with other studies^30^ we decided to check manually all matches and exclude the questionable taxa from the analyses (Table S1). The need for the manual check of the combined dataset is additionally enforced as, contrary to the COI, the V9 region of 18S does not ensure species-level detection and is more appropriate for genus- or even family-level detection^28^. Low barcoding resolution of this marker also became evident within our dataset, as many 18S assigned taxa were considered as a plausible misidentification (Table S1).

The groups of sampling sites inferred from the clustering analysis that separated coastal from offshore samples are in accordance with the dominant surface current pattern of the Adriatic Sea (Fig. 4) during the summer period. In general the Adriatic surface circulation is a cyclonic circulation formed by the Eastern Adriatic Current (EAC) (warm and salty) flowing northward along the eastern coast and the Western Adriatic Current (WAC) (intense and less salty) flowing southward along the western coast^35^. In spring and summer, the Po and other river discharges on the north-western coast spreads over large areas of the Northern Adriatic, developing semi-permanent cyclonic and anticyclonic subregional gyres reducing the water exchange with the central Adriatic^35^.

Species diversity, as depicted by Simpson’s diversity, did not show any clear pattern linked to the spatial distribution of the species (or at higher taxonomic level). On the contrary, exploring the dataset limited to copepods only, it appears that the southernmost samples have higher alfa diversity values due to the contribution of a large number of oceanic species opposed to the northern areas which are on average hosting less copepod diversity (Fig. 5); a pattern also in agreement with Hure *et al*.^27^.

The comparison between morphological and molecular identification provided very interesting insights, revealing strengths and weaknesses of these two approaches, and confirming that the molecular approach is not ready to replace a classical morphological inspection (Fig. 7A,B). As highlighted in other similar studies^16,36^ we found that the principal limitation for the molecular approach is the low number of sequences available for marine zooplankton indigenous species in genetic repositories. Looking at copepods, of the 12 species of the genus *Oithona* reported for the Adriatic, only the COI sequences of *O. similis*-group is deposited in the GenBank repository. Similarly, for the genus *Diaixis*, the only species recorded in the Adriatic, *D. pygmaea*, has never been barcoded. As this problem is affecting most basins, there are several ongoing dedicated barcoding projects (e.s.: BOLD and CmarZ) aiming at filling those gaps. On the other hand, the taxa identified in this study, exclusively by the molecular approach (Fig. 7A,B, Table S2) were often larval or juvenile stages of metazoans in zooplankton, which are notoriously difficult to be recognized by morphological characteristics. Other particular cases of exclusive identification through molecular barcoding were related to the presence of cryptic species (as in the case of the copepod genera *Oncaea* and *Paracalanus*). In this respect, it is worth highlighting the molecular detection of the copepod *Paracalanus quasimodo*, a species never reported before in the Adriatic but recently identified in the western Mediterranean^37^ and Black Sea^38^. All previous testimonies of *P. quasimodo* in the Mediterranean were considered a misclassification of congeneric *P. parvus* or *P. indicus*^37,39^, due to the large number of similar diagnostic characters. To make things more complex, other genetic studies have shown that there are multiple phylogroups that do not always correspond to morphotypes reported for *P. parvus* and *P. indicus*^37^. Our reads matched with sequences of specimens collected in the south-western Mediterranean by Cornils & Held^37^ at high degrees of similarity (97% for both, COI and 18S). Our results indicated the presence of this complex of species in the Adriatic, however it was not in the scope of this study to try to resolve any taxonomy dispute.

Another valuable help provided by a DNA metabarcoding approach is the potential in early detection and monitoring of expansion of non-indigenous species^15,22,40^. The detection of rare species represents technical challenges in all environments, but particularly in aquatic ecosystems^9,41^. However, especially in the case of 18S, extreme cautiousness should be applied due to the low resolution power of this marker and taxonomic assignments should in many cases be considered as a closest match rather than as a definite taxon. Therefore, to limit to the minimum false identifications of NIS (and other taxa as well) these assignments should be evaluated by expert taxonomists according to the knowledge of local distributions and also incorporating information from the COI marker and morphological identification (see below and also Table S1). Such an evaluated example is the Indo-Pacific copepod *Pseudodiaptomus marinus*, which was found for the first time in the northern Adriatic in 2007 near Rimini and later, in 2009, in the harbour of Monfalcone^42^. Data obtained in this study both with COI and 18S sequences and by morphological identification reconfirmed the presence of *P. marinus* in the Adriatic Sea and showed that this species enlarged its distribution as of September 2014 (present study) it was present in most of the northern basin and even at one coastal station in the southern Adriatic. The highest abundance of this species outside some of the major port areas in Northern Adriatic (Fig. 6A) supports the hypothesis in which its presence was associated to discharges of ship’s ballast water^43,44^.

The DNA metabarcoding approach has also proven to be helpful for monitoring the presence of early life stages (eggs and larvae) of commercially valuable species. Our results showed that areas for anchovy identified by molecular approach are in agreement with locations already known for this species: in the Gulf of Trieste (stations 33, 35) and several other sites in the northern and central Adriatic^29^. Of particular interest is the confirmation of a high abundance of early life stages of anchovies also in the southern Adriatic, in proximity of the Tremiti islands (sample 82^29^).

The study carried out in the Adriatic Sea confirms that DNA metabarcoding is providing an excellent platform for routine applications such as monitoring programmes, especially if extended to basin scale. HTS platforms combined with specifically designed pipelines are able to provide a large amount of information in relatively short time and at affordable prices for several samples at once. Considering a sampling campaign comparable to ours with the pipelines (molecular and bioinformatic) we have followed, the DNA metabarcoding dataset can be provided within more or less a month of work, while the screening of all the samples under the stereo microscope can occupy an expert for almost one year. This approach might well apply to fulfil the requirements of European legislation relating to the marine environment such as the Marine Strategy Framework Directive as well as providing information on the status of the marine environment. Nevertheless, DNA metabarcoding applied to zooplankton communities is not ready to replace traditional taxonomy, on the contrary the two approaches should be combined to take advantage in improving our knowledge of marine biodiversity.

## Material and Methods

### Sample collection

Sampling was carried out between August 27^th^ and September 16^th^, 2014, thanks to the survey supported by the MEDIAS project and the Italian Flagship project “Ritmare”. We monitored 46 stations in order to cover the northern and western Adriatic Sea between 42° and 46° of latitude (Fig. 3 with details in Table S3). Each haul was performed with a 200 μm mesh WP2 plankton net from 3 m above the bottom to the surface (or from 100 m in case of deeper waters). Sampling took place at different times within the 24 hours (Table S3).

Each plankton sample was divided into two parts: one part (half sample was immediately fixed in 4% buffered formalin to fix the sample for assessment using a light microscope) devoted to taxonomical analysis and the other half was preserved in 95% ethanol.

### DNA extraction and PCR amplifications

Genomic DNA extraction was performed using the E.Z.N.A.® Mollusc DNA kit (Omega Bio-Tech) following the manufacturer’s instructions. Prior to extraction, all samples were centrifuged at 3,200 g for 4 minutes to pellet the zooplankton and discard most of the ethanol in order to process 75–125 mg of pellet.

Quality and quantity of the extracted DNA was assessed with a NanoDrop 2000 Spectrophotometer (ThermoScientific). For each sample individual PCR amplifications were performed in two steps: a first reaction aiming to the amplification of a short fragment (~313 bp of the COI and ~138 bp for the 18S), followed by a second reaction to ligate proprietary adaptor sequence (P1) and unique 10–12 bp long identifier nucleotide key tags (Barcodes) compatible with the PGM Ion Torrent (Life Technologies) emulsion PCR for bioinformatic differentiation of pooled samples. In the first PCR amplifications, for the COI fragment, we used a cocktail of degenerated primers: mlCOIintF, dgHCOI2198 and jgHCOI2198^45^; while for the 18S, we employed the combination of 1391 F and for bioinformatic differentiation of pooled samples EukB^20^. All primers were modified at their 3′-ends adding a short tail of 18 nucleotides in length that will be used as anchorage for the second PCR to ligate barcodes and P1 labelling to the PCR product.

PCR amplifications (COI and 18S) were performed in a total volume of 12 μl with 0.60 μl of 10 μM of each universal forward and reverse primers, 5.0 μl of KAPA HiFi HotStart ReadyMix (KAPA Biosystems), 0.5 μl of EvaGreen dye (Biotium) and 1 μl of genomic DNA (<50 ng/μl).

The thermal cycling profiles started with 95 °C for 1 min. followed by 5 cycles of denaturation at 95 °C for 10 s, annealing at 46 °C for 20 s, extension at 72 °C for 10 s, and followed by a additional 23 or 21 cycles (for COI and 18S, respectively) of denaturation at 95 °C for 10 s, annealing at 54 °C for 10 s and extension at 72 °C for 10 s, with a final extension at 72 °C for 3 min.

The second PCR amplification (switch PCR) was performed using the same volumes of reagents as in the first PCR, but replacing the primers cocktail with Barcodes and P1 solution of 10 μM each and using 1 μl of the first PCR product (COI or 18S) as template. The thermal profiling for the switch PCR started at 94 °C for 3 min, followed by 10 cycles of denaturation at 94 °C for 10 s, annealing at 60 °C for 10 s and extension at 72 °C for 10 s, with a final extension at 72 °C for 3 min. Quantity and quality of the PCR products were determined with the Agilent DNA High Sensitivity Kit on the Bioanalyzer (Agilent) following the manufacturer’s recommendations.

### PGM Ion Torrent sequencing

Prior to the emulsion PCR, an equal amount of all COI products were pooled and processed for fragment size selection (range 150–400 bp) using E.Z.N.A.® Size Select-IT kit (Omega Bio-Tech). The quantity of DNA was assessed using a NanoDrop 2000 Spectrophotometer (ThermoScientific). Normalization for the 18S products was performed with the E.Z.N.A.® Mag-Bind Normalizer kit (Omega Bio-Tech) along with a magnetic separation device.

Amplicons (COI and 18S) were then subjected to emulsion PCR using the Ion One Touch (Life Technologies) following the manufacturer’s recommendations. For clonal amplification, DNA was localized to Ion Sphere particles (Life Technologies), which were automatically enriched with the Ion OneTouch ES system (Life Technologies).

Mass-parallel sequencing was carried out using the PGM (Life Technologies). Before initializing the PGM Sequencer, we performed a cleaning protocol that started with a chlorite cleaning solution and was followed by a wash with 18 MΩ water. After initialization, the chip was washed with 100% isopropanol and annealing buffer (from sequencing kit) and then tested for its functionality on the PGM. Sequencing primer and Control Ion Spheres of the Ion PGM sequencing kit were added to the library. After the annealing step sequencing polymerase was added and the sample was loaded onto the chip (316™ for COI and 314™ for 18S).

### Sequence data processing, taxonomic assignments of the mesozoplankton samples

Raw COI and 18S reads were demultiplexed, truncated (tags and primers) and processed using the *split_libraries.py* script from QIIME 1 v. 1.9.0 pipeline^46^ allowing three nucleotide mismatches in primers, while all other parameters were left as by default. The processing stage also included removal of low quality (minimum average Phred quality score >20) and short sequences (<200 bp or <100 bp for COI and 18S, respectively).

Taxonomic assignment of the COI and 18S dataset was done by aligning the individual reads against an in–house reference databases with a naive LCA-assignment algorithm implemented in the MEGAN6 alignment tool (MALT^47^) and using the similarity thresholds of 90%, 95% and 97% for both genes and 99% only for the 18S (Table S1); the two search queries in the GenBank database included all the metazoans except those groups of animals not present among marine zooplankton (for details see Figure S11). All datasets with the assigned taxonomy were manually checked and each assignment was placed in one of four categories in accordance with the known distribution of the corresponding taxa in the Adriatic Sea: taxon present in the Adriatic; taxon that was never recorded and is unlikely to be found in the Mediterranean Sea (erroneous assignments); taxon that has never been recorded in the Adriatic and is unlikely to be found in the Mediterranean, however a known closely related species (from the same genus or family) is recorded in the Adriatic (plausible misidentification); a known non-indigenous species (NIS) previously recorded in the Adriatic. In accordance with these evaluation, both erroneous and taxonomic assignments to single reads (singletons) were excluded from further analyses (see Figure S11).

In addition to individual taxonomically assigned datasets, a combined dataset (COI + 18S) was built by merging the COI-95% and the 18S-97% matrices – these thresholds were chosen in accordance to the different power of resolution of the two markers^48^. Prior to merging, the two matrices were adjusted for difference in sampling depth (mainly due to the different type of sequencing chips used), and stations that were not successfully sequenced by both markers were deleted (16, 43, 46). The adjustment weight of 3.7 was calculated from the proportion of the reads that passed quality filtering for both genes; after the weight adjustment the number of reads was rounded up. Furthermore, the combined matrix was polished manually, so in cases of double entries (taxonomical units retrieved by both reference databases) only the COI values were kept.

To compare the performance of the two markers in terms of taxonomy assignment a stepwise assignment procedure over a series of similarity thresholds decreasing of 1% at the time (from 100% to 70% for COI and to 80% for 18S) was conducted. Comparison of assigned taxonomy of the two markers was done at phylum level and the minimum percent similarity limit to discriminate between metazoan phyla with the two markers were defined as the similarity thresholds below which the reads started to become assigned to more than one phylum. For this purpose we built a new reference database including sequences of all eukariotes (not only metazoans) deposited in GenBank dataset.

### Clustering into molecular operational taxonomic units and community analysis

To explore the community diversity of both datasets a taxonomy free clustering into MOTUs approach was also applied. Reads from all samples were pooled pooled and de-replicated globally in QIIME 2 v. 2018.2^50^ and chimeras and “borderline chimeras” were removed with the VSEARCH algorithm^25^ using uchime-*de novo* approach. The same algorithm was also used for *de novo* clustering at 90, 95 and 97% dissimilarity level for COI reads and at 90, 95, 97 and 99% dissimilarity level for 18S reads. All the resulting MOTU tables were curated with the LULU algorithm^26^ to remove erroneous MOTUs using R software^50^. Details on scripts executed can be found in a workflow diagram presented in Figure S12 of the supplementary material section.

In addition to the above *de novo* MOTU datasets and the taxonomically assigned database we also constructed a set of “only metazoa” databases. As the primers used for the PCR amplifications of both markers are not unique to metazoans only, but also amplify other eukaryotes and even prokaryotes^21^, the original MOTU databases was polished to retain what are mostly likely to be only metazoans. These metazoan databases were constructed by extracting the reads assigned to the metazoan taxa at the minimum percent similarity limit evaluated above – 80% for COI and 86% for 18S (see Results and Figure S3). These threshold values were selected as the lowest limit for an assignment to phylum level. As the number of assigned reads started to level off below these limits we most likely retained a great majority of metaozan reads with this approach. The extracted reads were then subjected to the same *de novo* MOTU approach with QIIME2 outlined above, also clustering at 90%, 95% and 97% for both genes and 99% only for 18S and again curating with the resulting metazoan only MOTU tables with the LULU algorithm.

Finally, diversity assessments of all datasets was done with R software. Alpha diversities of individual communities were quantified using the iNEXT package^51^ and according to the measure of two Hill numbers – MOTU/taxa richness^52^ (q = 0) and Simpson’s diversity (q = 2, Gini–Simpson index^53^). Diversity estimates and the associated 95% confidence intervals were computed from a sample-size-based extrapolation of sampling curves and by estimating asymptotes^52^. Beta diversity was evaluated from dissimilarity matrices built according to Bray-Curtis distances (square root standardization) and visualised with NMDS using the metaMDS function from the vegan package^54^ and with the Ward’s hierarchical clustering method using ecodist^55^ and pvclust packages^56^, where stability at each node was assessed by an Approximately Unbiased test (AU) and Monte Carlo subsampling technique (1,000 permutations) in order to obtain empirical *P*-values. A separate clustering analysis was also performed just on reads assigned only to copepods. For the taxonomically assigned datasets taxa contribution to the differences between cluster and subclusters was analysed using the software package PRIMER6^57^, utilising the SIMPER (Similarity percentage) test.

### Taxonomic composition and abundance

Morphological analysis were performed on 8 samples: 24, 35, 56, 60, 62, 73, 76 and 82. Formalin-preserved samples were concentrated to remove the formaldehyde: samples were poured into a 200 µm sieve, washed in a round-bottom flask and made up to 200 ml with filtered seawater. After homogenization by mixing, 5 ml sub-samples were taken with a wide-bore pipette and examined under a stereo microscope (OLYMPUS SXZ12, up to 90x magnification) to identify the mesozooplankton community morphologically. The full sample was examined to identify and enumerate rare species. Copepods and cladocerans were identified at species level whenever possible while other groups, in particular meroplanktonic larvae, were mainly identified at higher taxonomic levels. Generally copepods were also distinguished to stage level (male/female/juvenile) and the eggs of the European anchovy (*Engraulis encrasicolus*) were identified and counted separately from other fish eggs.

Zooplankton abundances (ind m^−3^) were calculated using net mouth area and haul depth to estimate the volume of water filtered by the net.

## Electronic supplementary material

Supplementary figures S1-S11

Supplementary Tables S1-S3

## Data Availability

Accession codes of all PGM Ion Torrent generated sequences have been deposited in DRYAD (http://dx.doi.org/xxxxx).
