# Supplementary material for: Multi-marker metabarcoding approach to study mesozooplankton at basin scale
Source: Sci Rep. 2018 Aug 14;8:12085. doi: 10.1038/s41598-018-30157-7 (PMC6092319; doi:10.1038/s41598-018-30157-7)
Supplement: Supplementary file 1 — Supplementary figures S1-S11 [file 41598_2018_30157_MOESM1_ESM.pdf]

Supplementary Information for figures S1 – S11:

Multi-marker metabarcoding approach to study mesozooplankton at basin scale

Sergio Stefanni[a*], David Stanković[b, c], Diego Borme[d], Alessandra de Olazabal[d], Tea Juretić[d, e], Alberto Pallavicini[b], Valentina Tirelli[d].

Affiliations: [a]Stazione Zoologica Anton Dohrn, Villa Comunale, Naples, Italy; [b]Department of Life Sciences, University of Trieste, Via Licio Giorgieri 5, Trieste, Italy; [c]National Institute of Biology, Marine Biology Station, Fornače 41, Piran, Slovenia; [d]Istituto Nazionale di Oceanografia e di Geofisica Sperimentale (OGS), Via A. Piccard 54, Trieste, Italy; [e]Institute of Oceanography and Fisheries, Šetalište I. Meštrovića 63, Split, Croatia.

*corresponding author

E-mail: sergio.stefanni@szn.it

COI

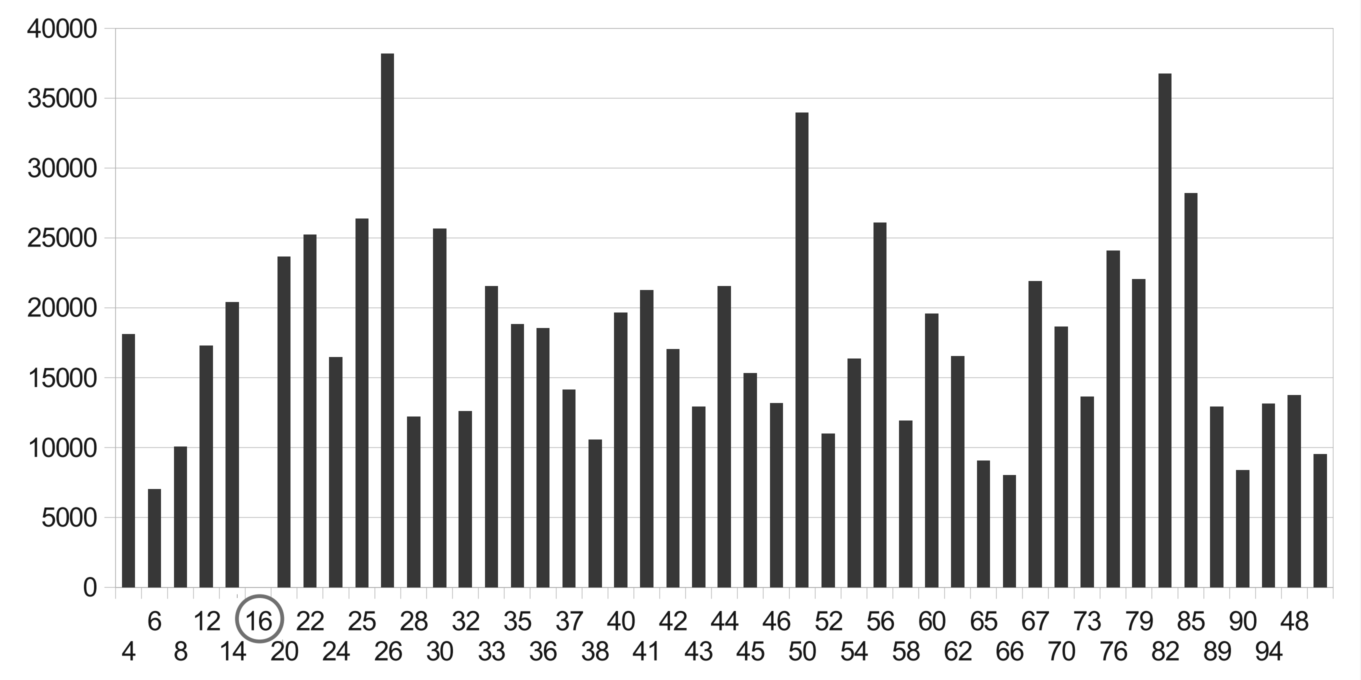

18S

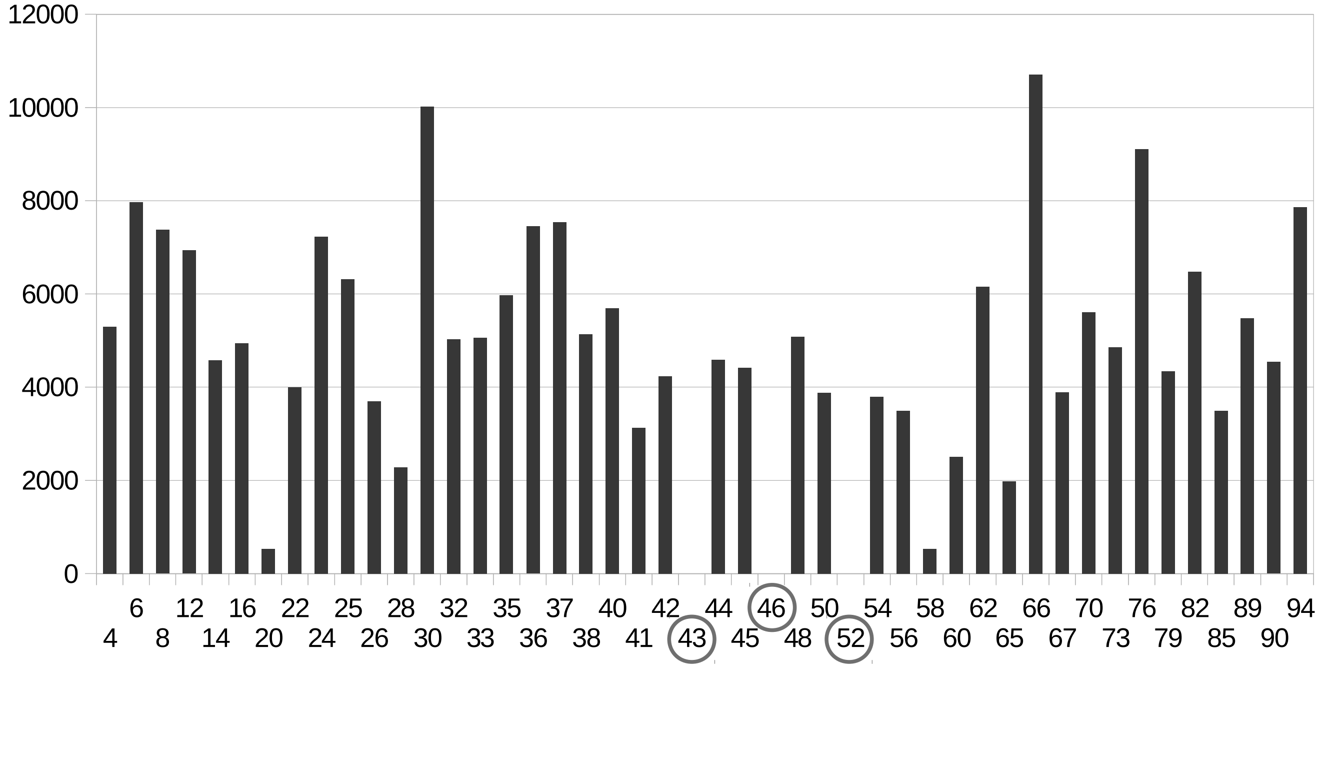

Figure S1. Distribution of reads for each sampling site for the two genes (COI and 18S). The samples highlighted with a circle did not produce any amplification and therefore were excluded in the analyses.

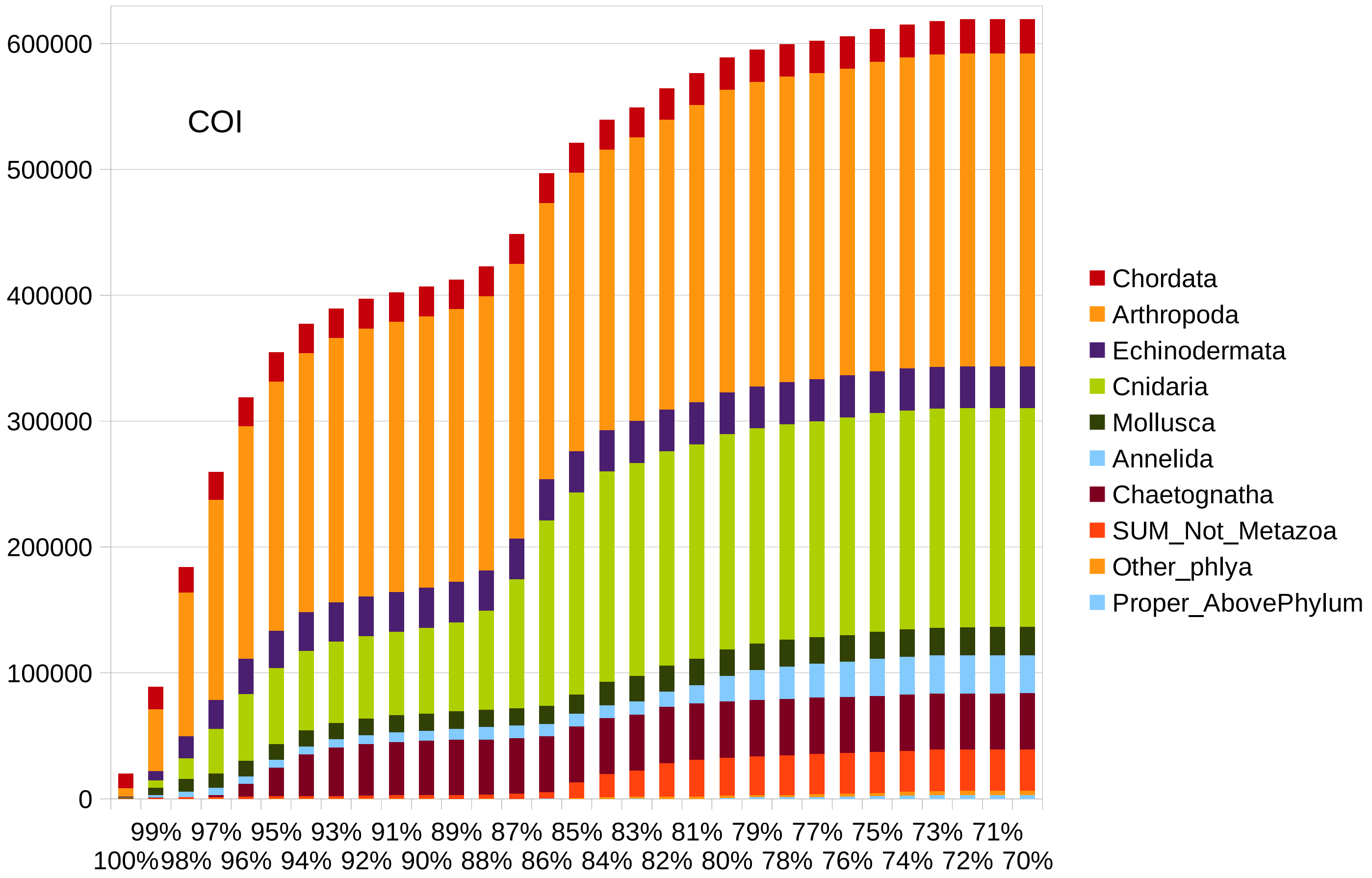

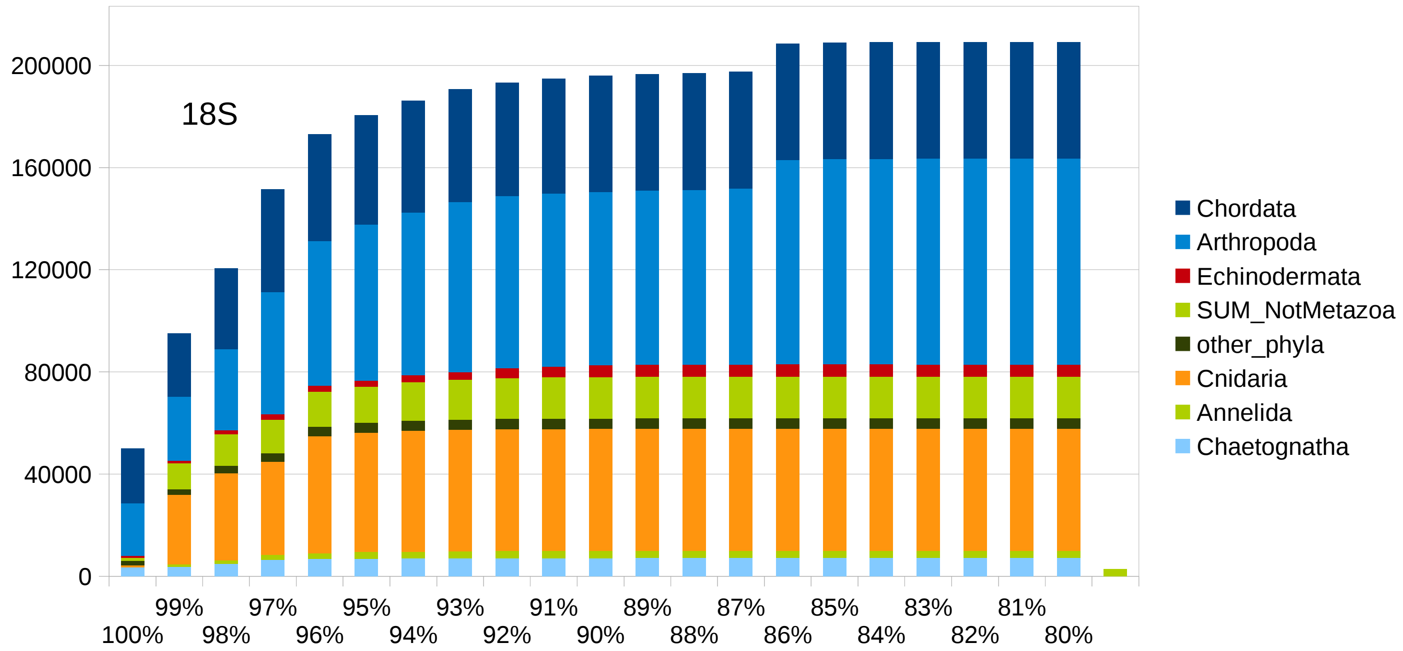

Figure S2. Bar plots showing the performance of individual molecular marker (COI and 18S) based on the number of reads assigned at each value of similarity thresholds.

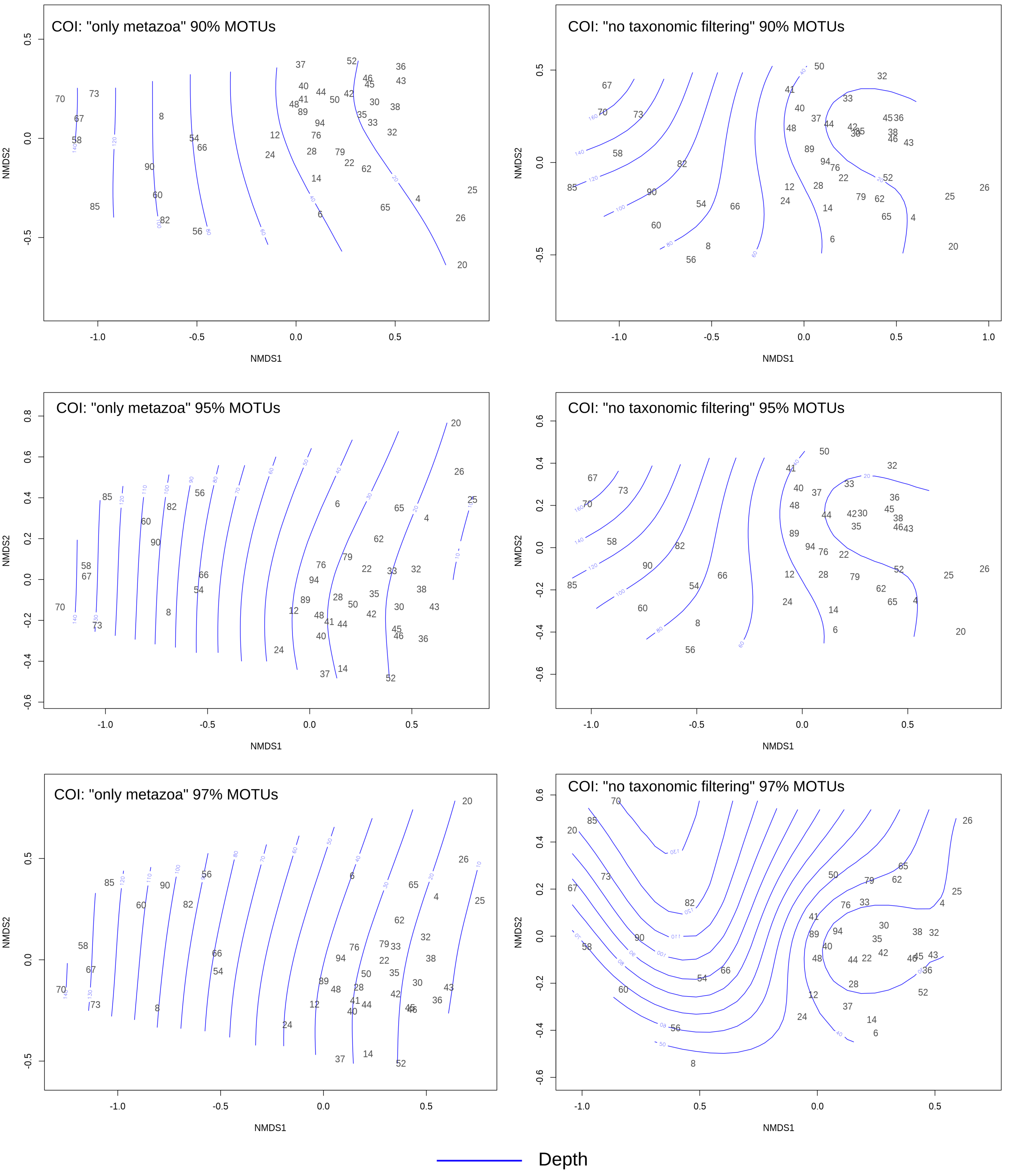

Figure S3. NMDS analyses of COI datasets depicting similarity in community composition based on abundance of MOTUs. Bathymetry in superimposed (blue contour lines) according to the depth measurements at the sampling sites (Table S3). The plots on the left side of the panel correspond to “only metazoa” while those on the right to “no taxonomic filtering” datasets. The plots from the top to the bottom correspond to different similarity levels used for MOTU clustering (90, 95 and 97% respectively).

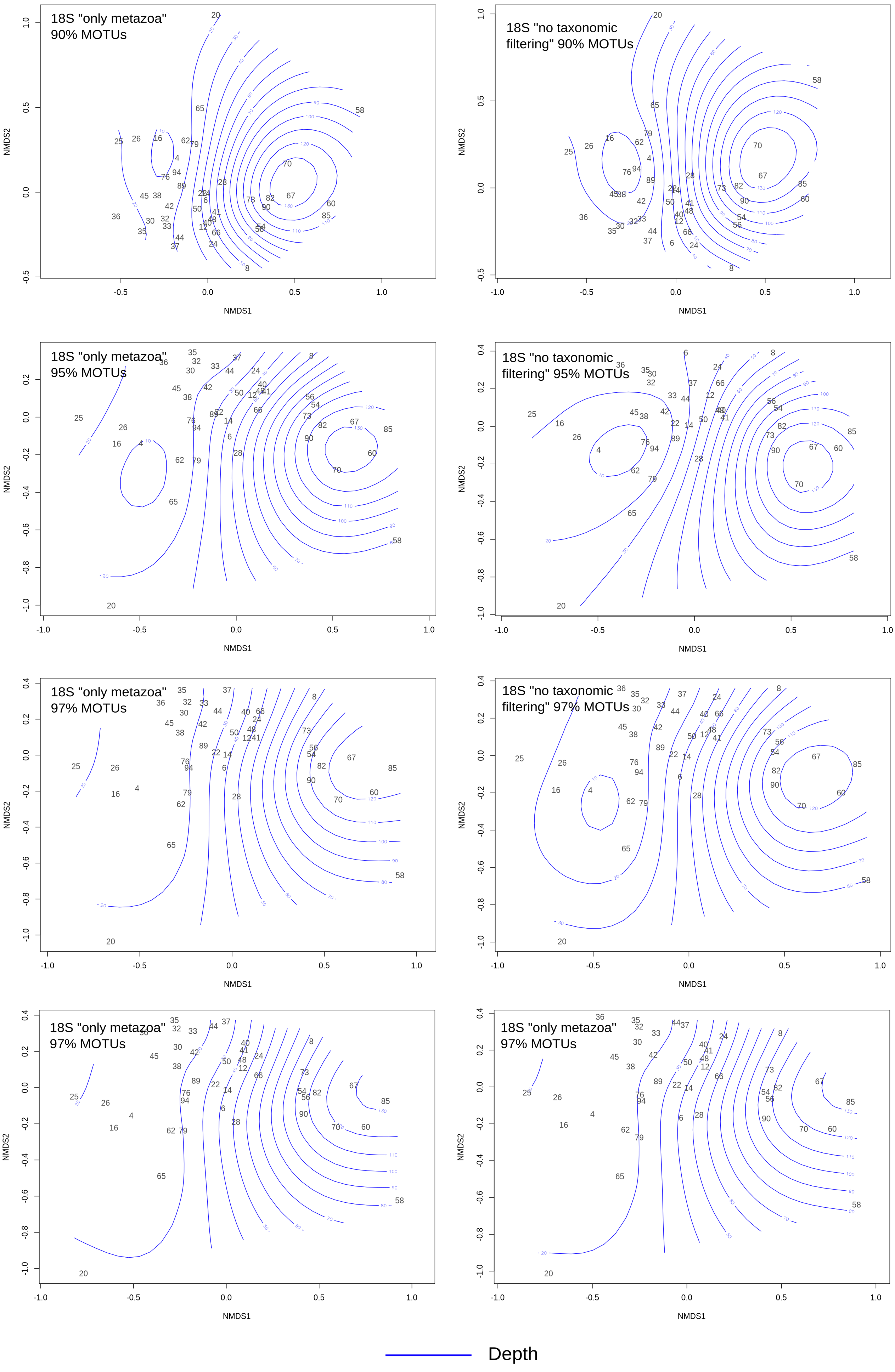

Figure S4. NMDS analyses of 18S datasets depicting similarity in community composition based on abundance of MOTUs. Bathymetry in superimposed (blue contour lines) according to the depth measurements at the sampling sites (Table S3). The plots on the left side of the panel correspond to “only metazoa” while those on the right to “no taxonomic filtering” datasets. The plots from the top to the bottom correspond to different similarity levels used for MOTU clustering (90, 95, 97 and 99% respectively).

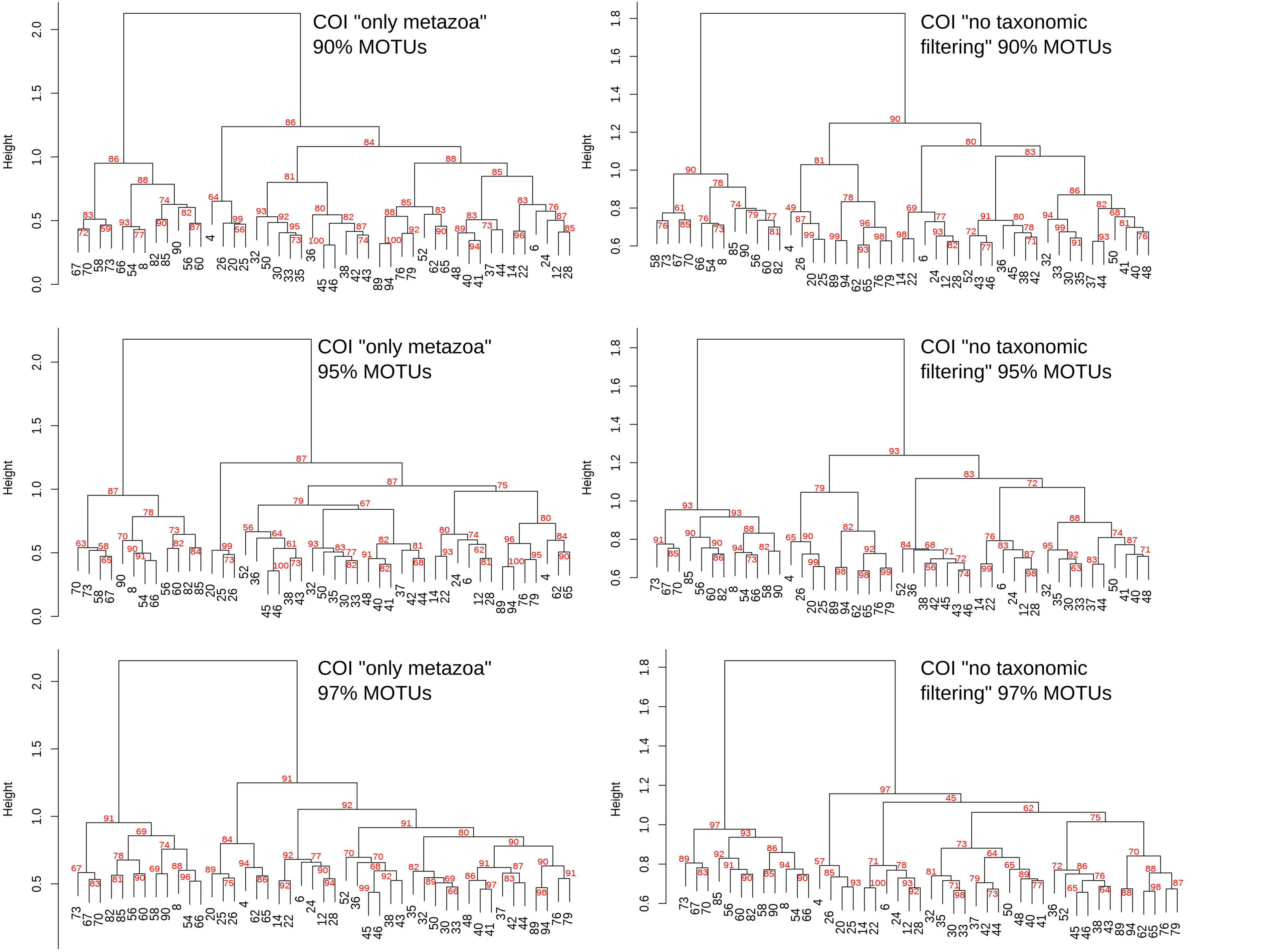

Figure S5. Clustering analyses of COI datasets depicting similarity in community composition based on abundance of MOTUs. The plots on the left side of the panel correspond to “only metazoa” while those on the right to “no taxonomic filtering” datasets. The plots from the top to the bottom correspond to different similarity levels used for MOTU clustering (90, 95 and 97% respectively).

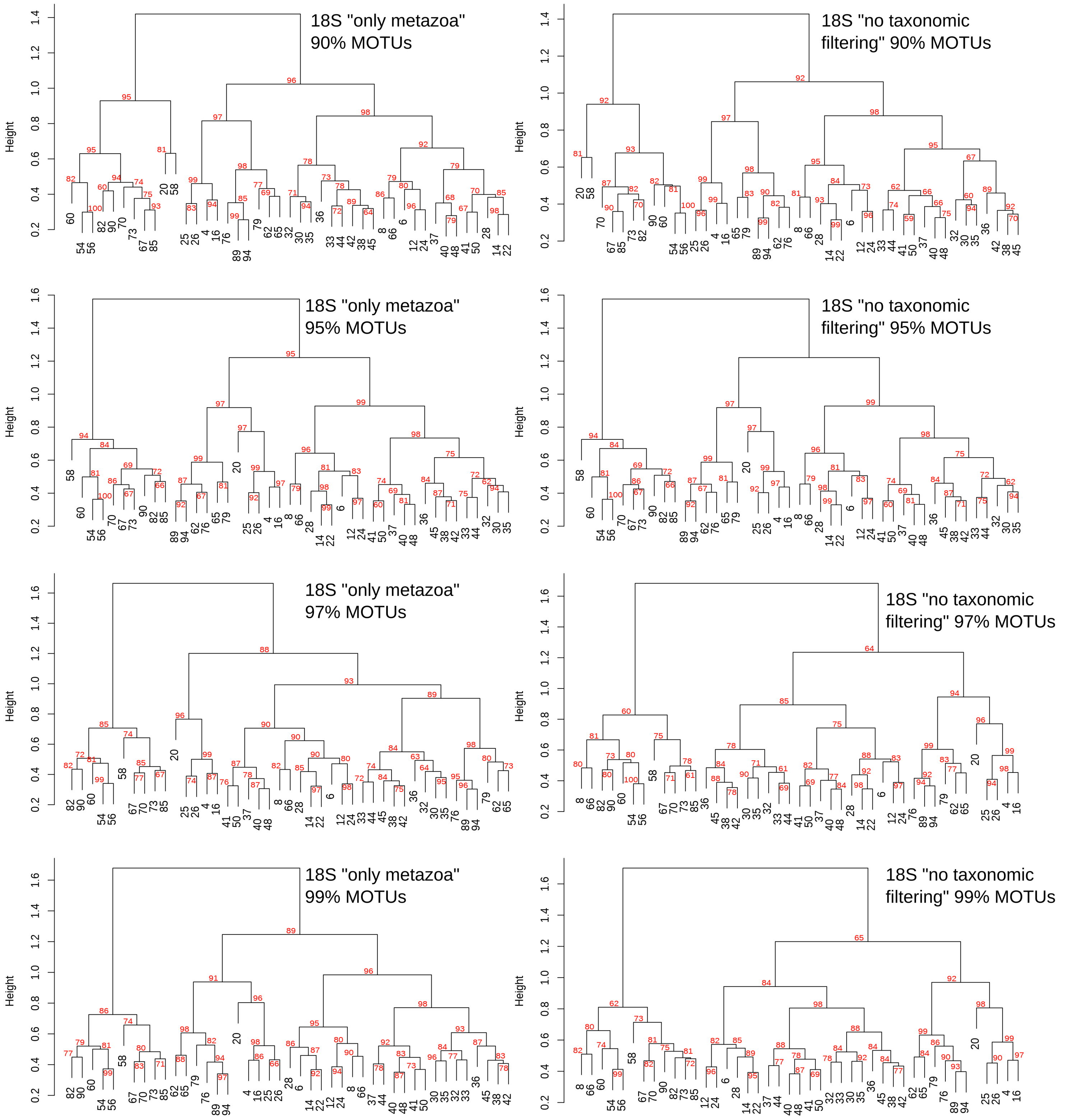

Figure S6. Clustering analyses of 18S datasets depicting similarity in community composition based on abundance of MOTUs. The plots on the left side of the panel correspond to “only metazoa” while those on the right to “no taxonomic filtering” datasets. The plots from the top to the bottom correspond to different similarity levels used for MOTU clustering (90, 95, 97 and 99% respectively).

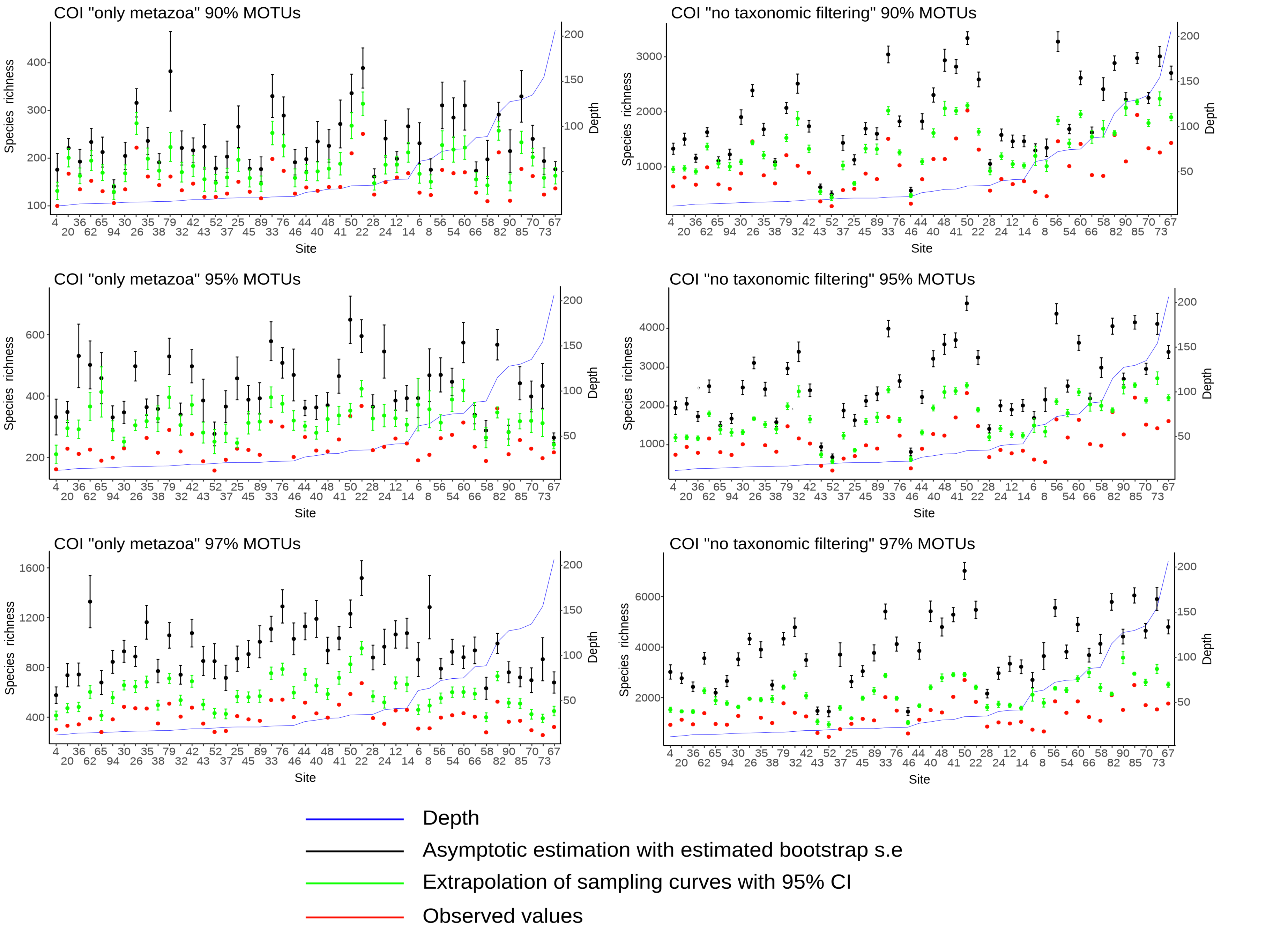

Figure S7. Sample-size-based estimation of species richness according to COI datasets. The plots on the left side of the panel correspond to “only metazoa” while those on the right to “no taxonomic filtering” datasets. The plots from the top to the bottom correspond to different similarity levels used for MOTU clustering (90, 95 and 97% respectively). The plot shows observed species richness (red), values estimated by extrapolating to the highest number of reads recorded in a sample with 95% CI (green), asymptotic estimation of species richness and standard errors estimated with bootstrap (black). In addition, bathymetry at sampling stations are presented by blue lines.

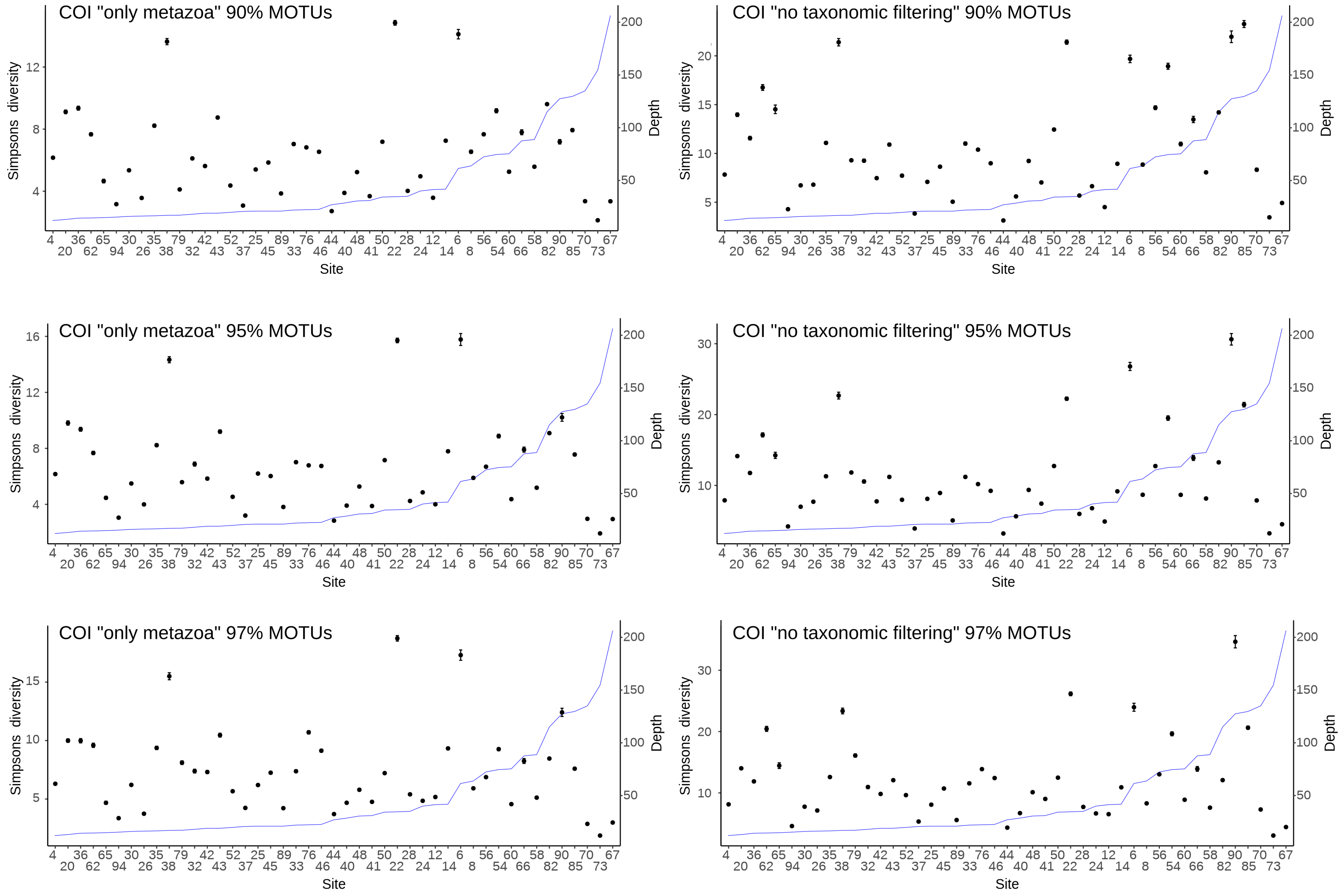

Figure S8. Sample-size-based estimation of Gini–Simpson index calculated from COI datasets. The plots on the left side of the panel correspond to “only metazoa” while those on the right to “no taxonomic filtering” datasets. The plots from the top to the bottom correspond to different similarity levels used for MOTU clustering (90, 95 and 97% respectively). Simpson’s diversity was estimated from asymptotes; error bars present standard error estimated by bootstrapping.

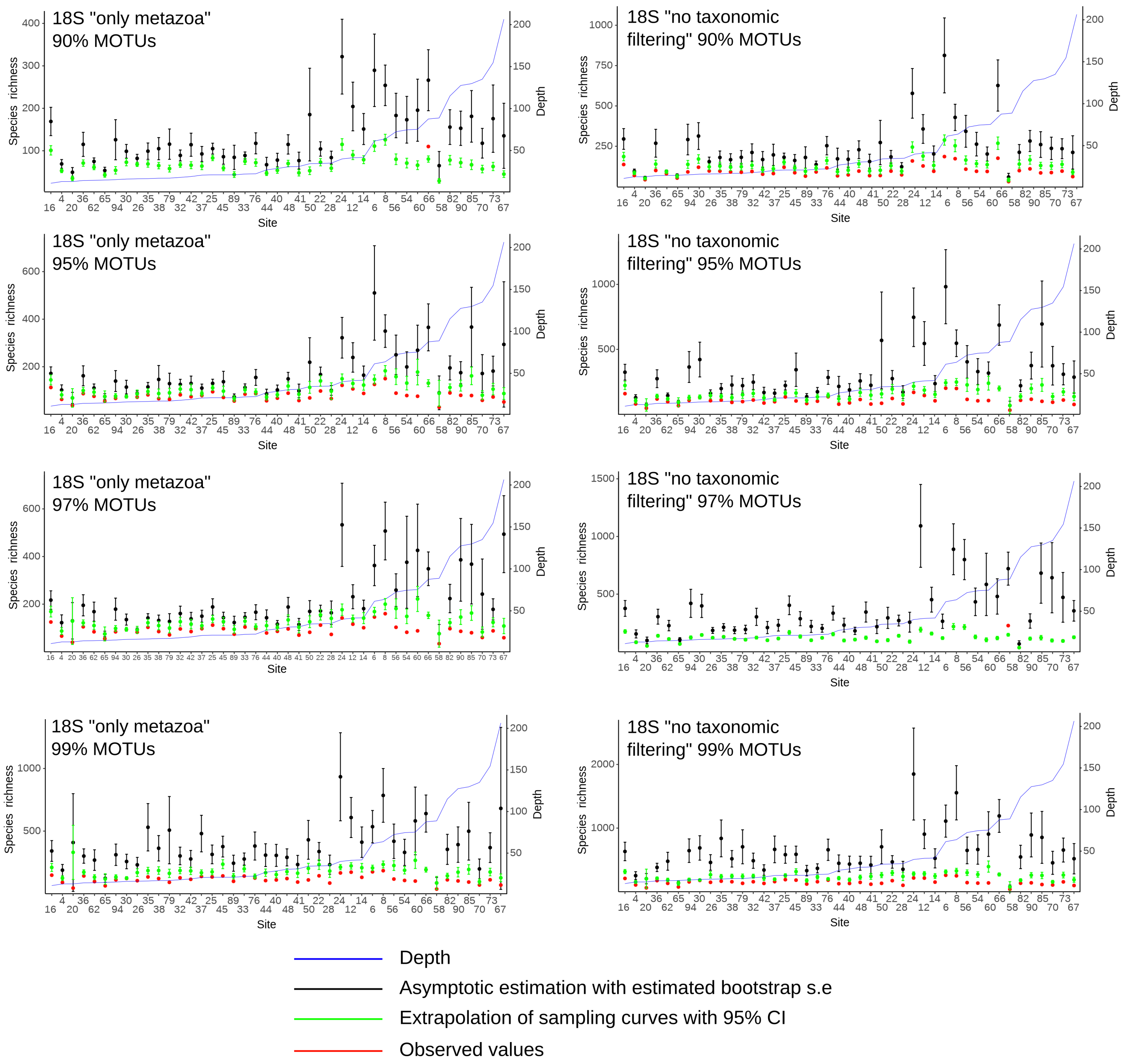

Figure S9. Sample-size-based estimation of species richness calculated from 18S datasets. The plots on the left side of the panel correspond to “only metazoa” while those on the right to “no taxonomic filtering” datasets. The plots from the top to the bottom correspond to different similarity levels used for MOTU clustering (90, 95, 97 and 99% respectively). The plot shows observed species richness (red), values estimated by extrapolating to the highest number of reads recorded in a sample with 95% CI (green), asymptotic estimation of species richness and standard errors estimated with bootstrap (black). In addition, bathymetry at sampling stations are presented by blue lines.

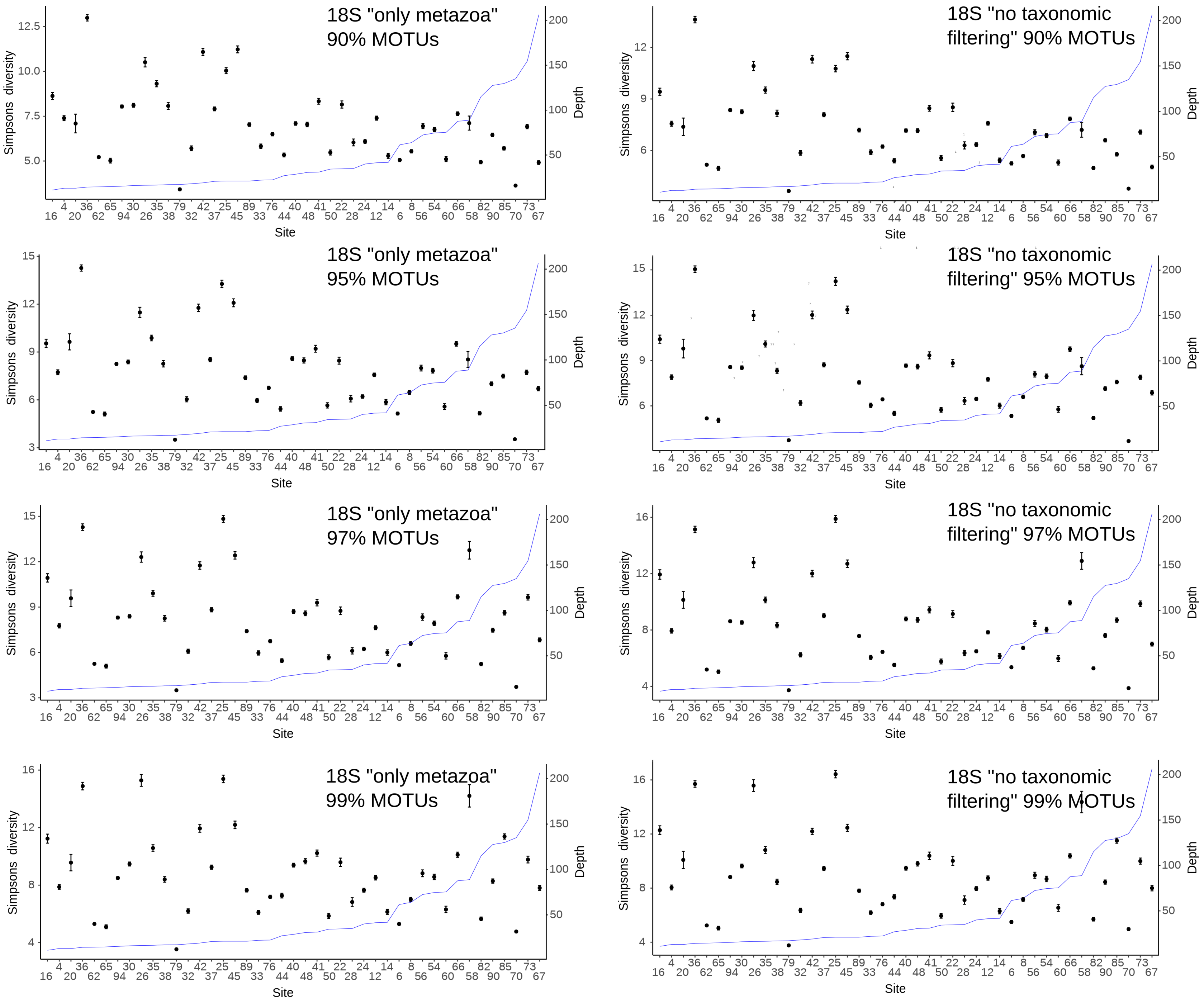

Figure S10. Sample-size-based estimation of Gini–Simpson index calculated from 18S datasets. The plots on the left side of the panel correspond to “only metazoa” while those on the right to “no taxonomic filtering” datasets. The plots from the top to the bottom correspond to different similarity levels used for MOTU clustering (90, 95, 97 and 99% respectively). Simpson’s diversity was estimated from asymptotes; error bars present standard error estimated by bootstrapping.

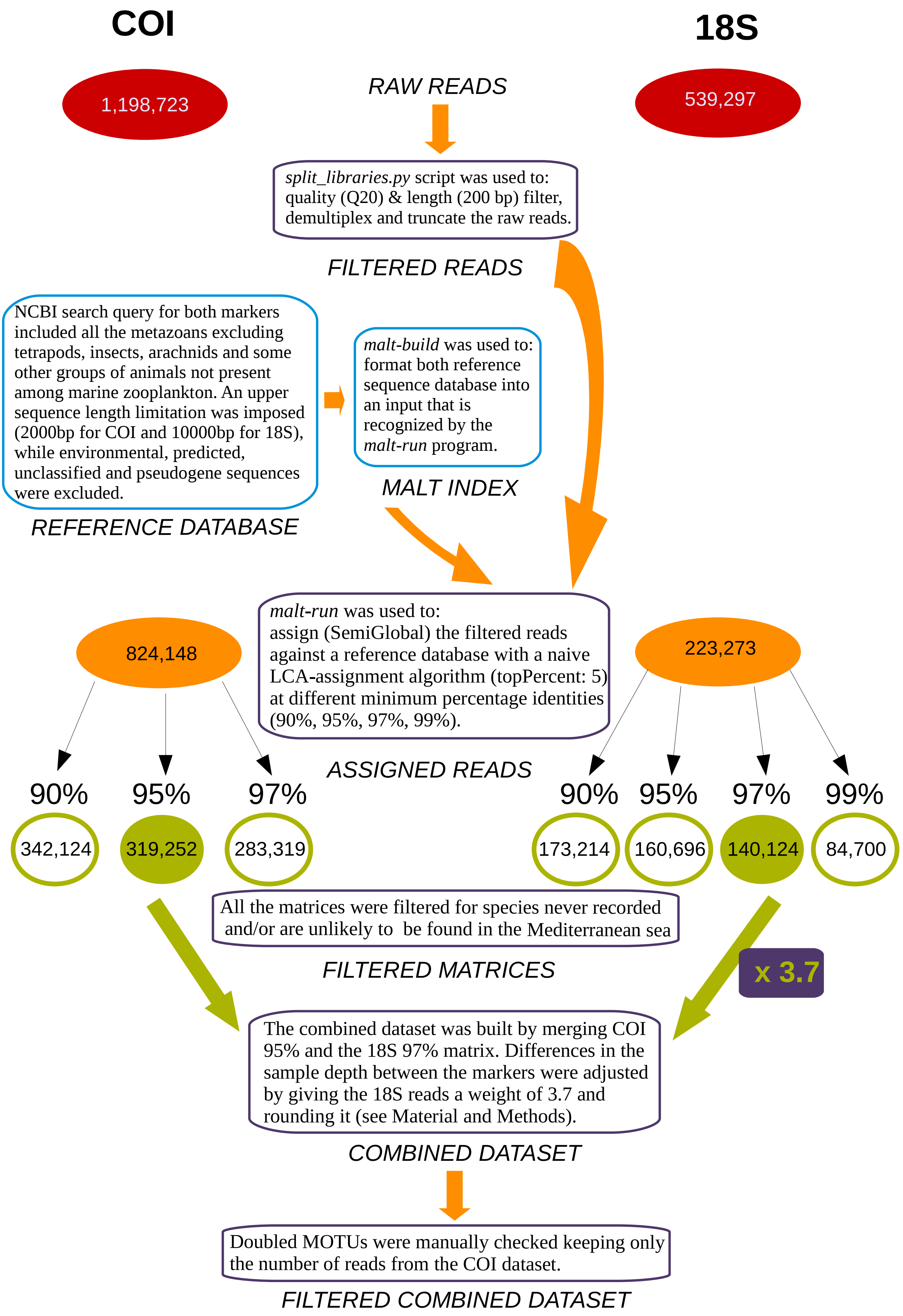

Figure S11. Flowchart showing the analytical steps applied on the two molecular markers (COI and 18S).
