# Supplementary material for: Multi-marker metabarcoding approach to study mesozooplankton at basin scale
Source: Sci Rep. 2018 Aug 14;8:12085. doi: 10.1038/s41598-018-30157-7 (PMC6092319; doi:10.1038/s41598-018-30157-7)
Supplement: Supplementary file 2 — Supplementary Tables S1-S3 [file 41598_2018_30157_MOESM2_ESM.pdf]

Supplementary Information for tables S1 – S3:

Multi-marker metabarcoding approach to study mesozooplankton at basin scale

Sergio Stefanni[a*], David Stanković[b, c], Diego Borme[d], Alessandra de Olazabal[d], Tea Juretić[d, e], Alberto Pallavicini[b], Valentina Tirelli[d].

Affiliations: [a]Stazione Zoologica Anton Dohrn, Villa Comunale, Naples, Italy; [b]Department of Life Sciences, University of Trieste, Via Licio Giorgieri 5, Trieste, Italy; [c]National Institute of Biology, Marine Biology Station, Fornače 41, Piran, Slovenia; [d]Istituto Nazionale di Oceanografia e di Geofisica Sperimentale (OGS), Via A. Piccard 54, Trieste, Italy; [e]Institute of Oceanography and Fisheries, Šetalište I. Meštrovića 63, Split, Croatia.

*corresponding author

E-mail: sergio.stefanni@szn.it

Phylum	Order	Family	0_NCBI TAXONOMY	COI 90%	COI 95%	COI 97%	18S 95%	18S 97%	18S 99%	Notes (WoRMS, WRIMS, Razouls, FISHbase, literature, personal knowledge)
Chaetognatha	Aphragmophora	Sagittidae	*Flaccisagitta enflata*	X	X	X	X	X	X	
			Parasagitta				X	X	X	18S identified it as either P.elegans or P.megalopthalma, of which neither is present in the Mediterranean
			Parasagitta setosa	X	X	X				
			Aidanosagitta crassa				X			
			Aidanosagitta neglecta				X	X	X	NIS
			Decipisagitta decipiens					X	+	
		Krohnittidae	*Krohnitta pacifica*				X	X	+	not in Med
	Elopiformes	Megalopidae	*Megalops atlanticus*				X	X	+	
Chordata	Anguilliformes	Congridae	*Bathyuroconger parvibranchialis*	X						Western Central Pacific
	Carangiformes	Carangidae	*Trachurus mediterraneus*	X	X	X				
	Pleuronectiformes	Pleuronectidae	*Rhombosolea tapirina*					X	-	not in Med (New Zealand)
		Soleidae	*Pegusa impar*	X	X	X				
			Buglossidium luteum		X	X				
	Perciformes	Cepolidae	*Cepola macrophthalma*	X	X	X				
		Osphronemidae	*Trichopodus trichopterus*				X			Asian freshwater
		Antennariidae	*Antennarius pictus*	X						Indo-Pacific
		Serranidae	*Serranus hepatus*	X	X	X				
		Sparidae	*Acanthopagrus butcheri*				X	X	X	
		Centracanthidae	*Spicara*	X	X	X				
	Gobiiformes	Gobiidae	*Aphia minuta*	X	X	X				
			Gobius niger	X	X	X				
	Atheriniformes	Atherinopsidae	*Atherinella sp. BOLD:AAZ7636*	X						not in Med
	Beloniformes	Belonidae	*Belone belone*	X	X	X				
	Syngnathiformes	Mullidae	*Mullus barbatus*	X	X	X				
	Clupeiformes	Clupeidae	*Sardina pilchardus*	X	X			X		
			Potamalosa richmondia					X	-	not in Med (Australia)
		Engraulidae	*Anchoa hepsetus*	X						Central America
			Engraulis encrasicolus	X	X	X	X	X	X	
			Engraulis australus		X					they might appear due to the highintraspecific variability of COI for Engraulis
			Engraulis japonicus		X					they might appear due to the highintraspecific variability of COI for Engraulis
			Engraulidae sp. BOLD:AAG4822	X						
Tunicata	Stolidobranchia	Styelidae	*Styela*					X		
			Styela plicata	X	X	X			+	NIS potential for Adriatic (Venice Lagoon)
	Salpida	Salpidae	*Thalia democratica*				X	X		
			Brooksia lacromae	X			X	X		
			Ritteriella retracta				X			
			Salpa maxima				X	X		
	Copelata	Oikopleuridae	*Oikopleura dioica*				X	X	X	
			Megalocercus huxleyi				X	X		not in Med
	Doliolida	Doliolidae	*Dolioletta gegenbauri*				X			
			Doliolum denticulatum				X	X		
			Doliolum nationalis				X	X		
Echinodermata	Euryalida	Gorgonocephalidae	*Gorgonocephalus eucnemis*							not in Med
		Ophiotrichidae	*Ophiothrix fragilis*	X	X	X	X	X		
			Ophiothrix oerstedii				X	X	-	not in Med (Gulf of Mexico)
		Ophiocomidae	*Ophiocoma echinata*				X	X	-	not in Med (Gulf of Mexico)
			Ophiopsammus angusta	X						New Zeland
		Ophiuridae	*Ophiocten sericeum*	X						not in Med. (Arctic Ocean, North Atlantic Ocean, Canada, West Coast of England)
			Ophioderma longicauda			X				
		Ophiomyxidae	*Ophiomyxa*				X			
			Ophiocanops fugiens							
		Amphiuridae	*Amphipholis squamata*				X	X		
			Amphiura abyssorum				X	X	+	Most likely A.filiformis
			Amphiura filiformis	X	X	X				
	Paxillosida	Astropectinidae	*Astropecten irregularis*	X	X	X				
	Clypeasteroida	Echinocyamidae	*Echinocyamus pusillus*				X	X	X	
	Spatangoida	Brissidae	*Brissopsis lyrifera*	X	X	X				
		Eurypatagidae	*Paramaretia multituberculata*	X						not in Med (Australia)
		Loveniidae	*Echinocardium cordatum*	X	X	X			+	
	Camarodonta	Parechinidae	*Paracentrotus lividus*	X	X	X	X	X		According to 18S this species is either P.lividus or Sterechinus neumayeri, however S.neumayeri is not Present in the Mediterranean

Phylum	Order	Family	0_NCBI TAXONOMY	COI 90%	COI 95%	COI 97%	18S 95%	18S 97%	18S 99%	Notes (WoRMS, WRIMS, Razouls, FISHbase, literature, personal knowledge)
			Psammechinus miliaris	X	X	X				not in Med
		Trigonocidaridae	*Genocidaris maculata*	X	X	X				
		Strongylocentrotidae	*Strongylocentrotus purpuraturs*							not in Mediterranean
	Aspidochirotida	Holothuriidae	*Holothuria*	X	X	X	X	X	X	
	Arbacioida	Arbaciidae	*Arbacia lixula*		X	X	X	X	X	
			Tetrapygus niger	X						not in Med (South Africa)
Hemichordata	Enteropneusta	Ptychoderidae	*Tergivelum*							
Platyhelminthes	Polycladida	Euryleptidae	*Prostheceraeus vittatus*				X	X		
Arthropoda	Pantopoda	Nymphonidae	*Nymphon australe*				X	X	+	not in Med (South Pacific)
		Pallenopsidae	*Pallenopsis macneilli*	X						not in Med (Australia)
	Diplostraca	Sididae	*Penilia avirostris*	X	X	X	X	X		
		Podonidae	*Evadne spinifera*	X	X	X	X	X		
			Evadne nordmanni							
			Pleopis	X	X	X				The MOTU is recognized as either P.polyphemoides or as P.sp. BOLD:AAI2700
			Pseudevadne tergestina	X	X	X				
			Podon intermedius	X	X	X	X	X	+	
	Decapoda	Penaeidae	*Melicertus kerathurus*	X	X	X				
			Parapenaeus longirostris	X	X	X				
			Penaeus japonicus				X			
		Sicyoniidae	*Sicyonia laevigata*				X	X	+	not in Med (Gulf of Mexico). Possibly S. carinata
			Solenocera membranacea	X	X	X				
		Aeglidae	*Aegla itacolomiensis*	X						Brazil freshwater
		Galatheidae	*Galathea acis*	X	X	X				not in Med
		Munididae	*Munida rutllanti*	X	X	X	X	X	+	
		Lithodidae	*Paralithodes camtschaticus*	X						not in Med
		Paguridae	*Paguridae sp. BOLD:ABW1528*	X	X					
		Dorippoidea	*Medorippe lanata*		X	X				
		Goneplacidae	*Goneplax rhomboides*	X	X					
		Calappidae	*Calappa granulata*	X	X	X				
			Calappa gallus				X			not in Med
			Cycloes granulosa							not in Med
			Hepatus epheliticus				X	X	-	not in Med
		Leucosiidae	*Ebalia cranchii*	X	X	X				
		Epialtidae	*Taliepus dentatus*	X						not in Med
		Inachidae	*Inachus dorsettensis*	X	X	X				
		Parthenopidae	*Parthenopoides massena*	X	X	X				
			Enoplolambrus validus							not in Med (China)
			Derilambrus angulifrons	X	X	X				
		Majidae	*Maja crispata*							
		Pilumnidae	*Pilumnus hirtellus*	X	X	X				
		Polybiidae	*Liocarcinus depurator*	X	X	X	X	X	X	
			Liocarcinus maculatus	X	X	X	X	X	X	
			Liocarcinus marmoreus	X	X					not in Med
			Liocarcinus pusilus	X						
		Portunidae	*Charybdis variegata*				X	X	-	not in Med (Subtropical. Indo-West Pacific: China, Taiwan and Japan)
		Xanthidae	*Xantho pilipes*	X	X	X				
			Ladomedaeus serratus				X			
			Micropanope pusilla					X	-	Gulf OF mexico; in MED M. Rufopunctata
		Erphioidead	*Eriphides hispida*					X	-	not in Med
			Cyclograpsus cinereus					X	-	not in Med (Pacific)
		Varunidae	*Gaetice depressus*				X	X	-	not in Med (Pacific)
			Hemigrapsus				X	X		According to 18S this species is either H.penicilatus or H.takanoi, but neither is present in Med. However, H.sanguinesu is a NIB confirmed in the Adriatic (Schubart, 2003) and its 18S sequence is missing in the NCBI database.
			Paragrapsus laevis				X	X		
		Macropthalmidae	*Macrophthalmus japonicus*				X	X	-	not in Med (Pacific)
		Ocypodidae	*Ocypode quadrata*				X	X	-	not in Med
		Alpheidae	*Alpheus*	X	X	X				COI recognize this MOTU as A.glaber, while 18S recognized both as A.lobidens and A.dentipes or just as Alpheus; A.glaber and A.dentipes are reported from the Adriatic, while A.lobidens from the Levantine coast. The NCBI database holds records of other species for both genes.
			Athanas nitescens	X	X	X	X	X		

Phylum	Order	Family	0_NCBI TAXONOMY	COI 90%	COI 95%	COI 97%	18S 95%	18S 97%	18S 99%	Notes (WoRMS, WRIMS, Razouls, FISHbase, literature, personal knowledge)
			Synalpheus tijou	X						not in Med (Australia)
		Hippolytidae	*Eualus cranchii*	X	X	X	X	X		
		Acanthephyridae	*Ephyrina figueirai*					X	-	
		Pandalidae	*Pandalus montagui*					X		not in Med (sub Arctic)
		Crangonidae	*Philocheras bispinosus*	X	X	X				
			Crangonidae sp. BOLD:ACR5265				X			
		Palaemonidae	*Palaemon*	X						
		Processidae	*Nikoides*	X			X	X	+	potential NIS: Genus not present in the MED; N. sibogae is ALIEN in the E. MED (Israel. 2014), where it was introduced from the Red Sea. The reference database is lacking both COI and 18S sequences from N. sibogae
			Processa modica	X	X	X				
			Processa nouveli	X	X	X				
		Laomediidae	*Jaxea nocturna*	X	X	X	X	X		
		Upogebiidae	*Upogebia acanthura*				X			not in Med
			Upogebia affinis				X			
			Upogebia deltaura	X	X	X				
	Euphausiacea	Euphausiidae	*Nematoscelis megalops*	X	X	X				
			Nyctiphanes australis	X	X					not in Med
			Nyctiphanes simplex	X						
			Thysanoessa				X	X		
			Thysanopoda aequalis				X	X		
	Stomatopoda	Squillidae	*Squilla mantis*	X	X	X	X	X	X	18S could not distinguish between S.mantis, S.empusa and Kempella mikado, however only S.mantis is Present in the Med
	Amphipoda	Hyperiidae	*Themisto japonica*	X						
			Themisto				X	X	X	
			Themisto abyssorum					X		
			Themisto gaudichaudii	X						
			Themisto libellula	X					X	not in Med
		Phronimidae	*Phronima bucephala*	X			X	X	+	
		Caprellidae	*Pseudoprotella phasma*							
		Oxycephalidae	*Cranocephalus scleroticus*							
			Streetsia challengeri				X			
		Aoridae	Aora gracilis				X	X		
	Mysida	Mysidae	*Anchialina agilis*				X	X		
			Gastrosaccus spinifer				X	X		
			Haplostylus dispar							
			Erythrops				X	X	-	
			Acanthomysis longicornis				X	X		
			Diamysis mesohalobia				X	X	+	
			Siriella							
			Paramysis				X	X	+	
			Bowmaniella sp. EC1				X	X	-	not in Med
	Calanoida	Acartiidae	*Acartia* (Acartiura) *clausi*	X	X	X	X	X		
			Acartia tonsa	X	X	X				
		Aetideidae	*Pseudochirella obtusa*	X						
		Calanidae	*Calanus helgolandicus*	X	X	X	X	X		
			Calanus euxinus			X				
			Calanus finmarchicus				X	X	+	
			Calanus jashnovi				X	X	+	
			Calanus sinicus					X	-	
			Ctenocalanus vanus	X	X	X				
			Mesocalanus tenuicornis	X	X	X		X	+	
			Nannocalanus minor	X	X	X				
		Calocalanidae	*Calocalanus contractus*	X	X	X				
			Calocalanus sp. 2 AC-2013	X						
			Calocalanus sp. 1 AC-2013	X						
		Candaciidae	*Candacia armata*				X	X		
		Centropagidae	*Centropages*				X	X	X	18S cannot distinguish between C.typicus, C.hamatus and C.abdominalis in all reads. While the first two Are present in the Med C.abdominalis is not.
			Isias clavipes				X	X		
		Clausocalanidae	*Clausocalanus arcuicornis*	X	X	X				

Phylum	Order	Family	0_NCBI TAXONOMY	COI 90%	COI 95%	COI 97%	18S 95%	18S 97%	18S 99%	Notes (WoRMS, WRIMS, Razouls, FISHbase, literature, personal knowledge)
			Clausocalanus furcatus	X	X	X				
			Clausocalanus jobei	X	X	X				
			Clausocalanus parapergens	X	X					
			Clausocalanus paululus	X	X	X				
			Clausocalanus pergens	X	X	X				
			Pseudocalanus minutus	X						not in Med
			Pseudocalanus elongatus	X	X	X		X		
			Pseudocalanus moultoni				X	X	+	not in Med
			Microcalanus sp. AC-2010				X	X	-	
		Diaixidae	*Diaixis hibernica*	X	X	X				
		Euchaetidae	*Paraeuchaeta hebes*	X	X	X				
		Metridinidae	*Pleuromamma gracilis*	X	X	X				
			Pleuromamma robusta				X	X	X	
		Paracalanidae	*Paracalanus aculeatus*	X						
			Paracalanus aff. indicus AC-2013	X	X	X				
			Paracalanus nanus	X	X	X				
			Paracalanus indicus		X	X				
			Paracalanus parvus	X	X	X	X	X		
			Paracalanus quasimodo	X	X	X				
		Pontellidae	*Pontella*				X	X	+	
			Anomalocera patersoni				X			
		Pseudodiaptomidae	*Pseudodiaptomus marinus*	X	X	X	X	X	+	NIS
		Lucicutiidae	*Lucicutia flavicornis*	X	X	X				
		Temoridae	*Temora*				X	X	X	
			Temora longicornis	X	X	X				
			Temora stylifera	X	X	X				
		Ridgewayiidae	*Exumella mediterranea*							
	Cyclopoida	Cyclopidae	*Tritia*	X	X	X				
			Diacyclops bisetosus							
		Oithonidae	*Oithona similis*	X	X	X	X	X		
			Oithona nana				X	X		
			Oithona davisae		X	X				
			Oithona simplex							
			Oithona sp. 1 New Caledonia-RJH-2001							
			Oithona sp. 2 New Caledonia-RJH-2004				X			
			Dioithona oculata				X			
	Harpacticoida	Euterpinidae	*Euterpina acutifrons*	X	X	X	X	X		
		Ectinosomatidae	*Bradya* sp. Greenland-RJH-2004							
		Canuellidae	*Canuella perplexa*				X	X	+	Transition waters
		Peltidiidae	*Peltidium* sp. New Caledonia-RJH-2007							
			Goniopsyllus rostratus	X	X	X				
	Poecilostomatoida	Oncaeidae	*Oncaea cf. mediterranea 'broad form'*	X	X					
			Oncaea scottodicarloi	X	X	X				
			Oncaea venusta	X	X	X		X		
			Oncaea waldemari	X	X	X				
			Oncaea sp. MVZ-2013				X	X	+	
			Triconia elongata	X	X	X				
			Triconia umerus	X	X	X				
		Corycaeidae	*Ditrichocorycaeus anglicus*	X	X	X				
	Siphonostomatoida	Pennellidae	*Lernaeocera branchialis*				X			
	Sessilia	Balanidae	*Amphibalanus amphitrite*	X	X	X				
			Amphibalanus improvisus	X	X	X				
			Amphibalanus sp. OPC-2014	X	X	X				not in Med
			Balanus trigonus	X	X	X				
		Chthamalidae	*Chthamalus stellatus*	X	X	X				
		Coronulidae	*Chelonibia testudinaria*	X	X	X				
		Pyrgomatidae	*Ceratoconcha domingensis*	X						not in Med
	Halocyprida	Halocyprididae	*Conchoecla sp. SN008*				X			not in Med
			Conchoecia sp. OC-2001				X			
			Archiconchoecia striata	X	X	X				

Phylum	Order	Family	0_NCBI TAXONOMY	COI 90%	COI 95%	COI 97%	18S 95%	18S 97%	18S 99%	Notes (WoRMS, WRIMS, Razouls, FISHbase, literature, personal knowledge)
			Metaconchecia skogsbergi	X						
			Porroecia spinirostris	X	X	X				not in Med
			Proceroecia microprocera	X	X	X				not in Med
	Diptera	Chironomidae	*Chironomus salinarius*	X	X	X				Ali et al. 1985 (J Am Mosq Control Asso)
Annelida	Eunicida	Eunicidae	*Eunice roussaei*	X	X	X				
			Marphysa sanguinea	X						
	Amphinomida	Euphrosinidae	*Euphrosine armadillo*					X	-	
	Phyllodocida	Phyllodocidae	Alitta succinea			X				
			Phyllodocidae sp. BMOO-13076	X						not in Med
			Phyllodoce groenlandica				X	X		
			Phyllodoce longipes					X	+	
			Pseudomystides bathysiphonicola					X		Not reported from Med.
			Glycera				X			
			Glycera alba					X		
			Eteone longa				X			
		Lopadorrhynchidae	*Pelagobia cf. longicirrata OK-2012*					X	-	
		Pilargidae	*Ancistrosyllis sp. AAN-2002*				X			not in Med (California)
			Sigambra sp. BC-2003				X	X		
		Nereididae	*Hediste diversicolor*	X	X	X				
		Polynoidae	*Austrolaenilla pelagica*	X						not in Med
			Lepidonotus					X	X	
		Tomopteridae	*Tomopteris*	X			X			
		Sigalionidae	*Sigalion spinosus*				X	X	+	
	Sabellida	Sabellidae	*Amphicorina*				X			
		Oweniidae	Owenia fusiformis	X	X	X	X	X		
		Sabellariidae	*Sabellaria alveolata*				X	X	+	
			Sabellaria spinulosa	X	X	X				
	Spionida	Spionidae	*Polydora cornuta*	X	X	X	X	X	X	According to 18S this is P.brevipalpa, while it is either P.haswelli (95%) or P.cornuta (95%, 97%) according to COI. While all the species are not naitve in the Med, only P.cornuta is reported as a NIS here (including Adriatic).
			Prionospio dubia				X	X	-	
			Spio sp. LK-2011-1				X	X	+	
			Spionidae sp. BMOO-13880	X						
			Spiophanes							
			Spiophanes kroyeri					X		
			Spiophanes bombyx				X			
			Boccardiella ligerica							
			Aurospio dibranchiata					X	-	
		Magelonidae	*Magelona sp VR-2004*				X	X	+	
		Poecilochaetidae	*Poecilochaetus serpens*	X	X	X	X	X	X	
			Poecilochaetus sp. VR-2006				X	X		
	Ophelida	Ophelidae	*Ophelina/Armandia*				X	X	X	
			Polyophthalmus pictus				X	X	X	
	Capitellida	Capitellidae	*Capitella capitata CMC02*	X						
		Maldanidae	*Asychis disparidentata*				X			not in Med
		Chaetopteridae	*Mesochaetopterus taylori*							
			Chaetopterus cf. *luteus* KJO-2005				X	X	-	
			Telepsavus sp. BVB-2002				X	X	+	
	Terbellida	Terbelidae	*Lanice conchilega*				X	X		
		Cirratulidae	*Tharyx sp. THS-2012*					X	-	
	Golfingiida	Sipunculidae	Sipunculus nudus	X	X	X	X	X		
Brachipoda			Phoronis hippocrepia				X	X		
	Lingulida	Lingulidae	Phoronis muelleri				X	X		
Bryozoa	Ctenostomatidae	Flustrellidridae	*Flustrellidra armata*	X						doubt in Med
Mollusca	Anomalodesmata	Cupispidariidae	*Cupispidaria cupispidata*	X	X	X				
	Myida	Corbulidae	*Corbula gibba*	X	X	X				
		Hiatellidae	*Hiatella*				X	X	+	
			Hiatella sp. D HML-2015	X	X	X				
		Placunidae	*Placuna placenta*				X	X		
		Teredinidae	*Lyrodus/Bankia*				X	X	X	According to 18S this MOTU is either Lyrodus pedicellatus or Bankia carinata. However, NCBI Database also holds COI sequences for both species.

Phylum	Order	Family	0_NCBI TAXONOMY	COI 90%	COI 95%	COI 97%	18S 95%	18S 97%	18S 99%	Notes (WoRMS, WRIMS, Razouls, FISHbase, literature, personal knowledge)
	Adapedonta	Pharidae	*Ensis magnus*	X						
	Veneroida	Veneridae	*Ruditapes philippinarum*	X	X	X		X	+	NIS
			Timoclea ovata	X	X	X				
		Pharidae	*Phaxas pellucidus*			X	X	X		
	"Imparidentia"	Montacutidae	*Kurtiella bidentata*	X		X				
		Gastrochaenidae	*Gastrochaena dubia*	X	X	X	X	X		
	Cardiida	Semelidae	*Abra alba*	X		X		X	X	
		Cardiidae	*Vasticardium*			X	X	X		
			Parvicardium minimum			X	X	X		
		Tellinidae	*Iridona iridescens*				X	X	X	*Not reported in Med*
	Arcida	Arcidae	*Anadara transversa*	X	X	X		X	+	NIS
			Tegillarca nodifera					X	-	
		Noetiidae	*Estellarca olivacea*					X	-	
	"Imparidentia"	Lasaeidae/Kellidae	*Lasaea/Kellia*				X	X		According to 18S this MOTU is either Lasaea rubra or Kellia cf.jacksoniana. While L.rubra is present in the Adriatic, the genus Kellia is not native in the Mediterranean; howere K.suborbicularis Is reported to be present in the Mediterranean as NIS.
		Montacutidae	*Mysella vitrea*			X	X	X		
			Tellimya ferruginosa			X	X	X	X	
	Limoida	Limidae	*Limaria hians*	X	X	X	X			
	Mytiloida	Mytilidae	*Mytilus*				X	X		
			Arcuatula senhousia					X	X	NIS
			Modiolus	X			X	X		According to COI this MOTU belongs to M.phaesolina, while 18S recognized it as either M.americanus, M.rufus or M.modiolus. Of these only the last species is reported for the Med and Is a potential NIS.
			Musculus discors / M. lateralis				X	X		
			Adula falcatoides					X	-	not in Med (Pacific)
	Ostreoida		*Hyotissa hyotis / H. mcgintyi*				X	X	+	*potential NIS*
		Gryphaeidae	*Neopycnodonte cochlear*	X	X	X				
		Ostreidae	*Crassostrea gigas*	X	X	X	X	X	+	NIS
			Ostrea edulis	X	X	X		X		
	Pterioida	Pinnidae	*Atrina fragilis*	X	X	X				
			Atrina pectinata	X						
	Pectinida	Pectinidae	*Chlamys hastata*					X	+	
			Mimachlamys varia				X	X	X	
	Sepiida	Sepiidae	*Sepia officinalis*	X	X	X				
	Teuthida	Loliginidae	*Loligo vulgaris*	X	X	X				
	"Sorbeoconcha"	Cypraeidae	*Cribrarula exmouthensis*	X						
		Dialidae	*Erronea rabaulensis*	X						
			Dialidae sp. BMOO-03134	X						
		Turritellidae	*Turritella communis*	X	X	X				
	Littorinimorpha	Litorinidae	*Echinolittorina punctata*					X	-	NIS
		Hydrobiidae	*Ecrobia maritima*	X	X	X				
		Pomatiopsidae	*Neotricula aperta*	X						Freshwater
	"Neogastropoda"	Conidae	*Conasprella mindana*	X						not in Med
			Conus pertusus	X						not in Med
			Mangelia attenuata	X						
		Muricidae	*Neothais harpa*	X						not in Med
		Mitridae	*Domiporta filaris*	X						not in Med
		Fasciolariidae	*Amiantofusus sebalis*				X	X		not in Med
	Cephalaspidea	Haminoeidae	*Haminoea orteai*	X	X	X				
			Atys semistriata					X	X	
		Retusidae	*Retusa umbilicata*	X	X	X				
		Aglajidae	*Chelidonura inornata*				X	X	-	
	Thecosomata	Cavoliniidae	*Cavolinia inflexa*	X	X	X				
			Creseis				X	X		
			Creseis clava	X	X	X				
			Creseis virgula	X	X	X				
			Diacria quadridentata					X	+	not in Med
		Cuvierinidae	*Cuvierina columnella*				X	X	-	
		Limacinidae	*Heliconoides inflatus*	X	X	X				
	Nudibranchia	Dendrodorididae	*Dendrodoris fumata*					X	-	NIS

Phylum	Order	Family	0_NCBI TAXONOMY	COI 90%	COI 95%	COI 97%	18S 95%	18S 97%	18S 99%	Notes (WoRMS, WRIMS, Razouls, FISHbase, literature, personal knowledge)
	Pleurobranchomorpha	Pleurobranchidae	Pleurobranchaea meckeli	X	X	X	X	X	X	
	“Hygrophila <snail>”	Lymnaeidae	*Lymnea stagnalis*	X						Freshwater
		Planorbidae	*Bulinus truncatus*	X						Freshwater
	“Pupilloidea”	Pyramidulidae	*Pyramidula pusilla*	X						Freshwater
	Sacoglossa	Limapontiidae	*Aplysiopsis minor*				X	X	X	Not present in Med. However, A.elegans was recently recorded in the Adriatic (Mavrič et al., 2014); A.minor (18S) and A.enteromorpha (COI) are the only species of the genus deposited in the NCBI Database.
			Ercolania viridis	X	X	X				
			Elysia sp. 3 GC-2014	X	X	X				
			Calliopaea bellula				X	X	+	not in Med
	“Acteonoidea”	Acteonidae	*Acteon tornatilis*	X	X	X	X	X		
	“Vetigastropoda”	Trochidae	*Diloma concamerata*	X						not in Med
Nemertea		Hubrechtidae	*Hubrechtella dubia*				X	X	X	
		Carinomidae	*Carinoma* sp. BOLD: ACM5178	X						
		Tubulanidae	*Tubulanus* sp. MCZ IZ 45559	X						
			Callinera grandis				X	X	+	not in Med
	Heteronemertea	Lineidae	*Micrura varicolor*	X	X	X				not in Med
			Micrura	X	X	X	X	X		According to COI this MOTU belongs to M.varicolor, while 18S recognized it as either M.ignea or M.fasciolata. Of these only the last species is reported for the Med, while other species of this Genus are also reported for the Med.
			Riseriellus occultus	X						
	Monostilifera		*Paranemertes*	X						
		Poseidonemertidae	*Poseidonemertes*				X	X		
Cnidaria	Antohoathecata	Zancleidae	*Zanclea* sp. ZX JRH-2014	X						
	Actinaria	Aliciidae	*Triactis producta*				X			
		Actiniidae	Anemonia sulcata				X	X		
		Balellidae	*Balella mirabilis*	X						not in Med
		Hydractiniidae	*Clavactinia serrata*							not in Med
		Bougainvillidae	*Bougainvillia muscus*	X	X	X		X		
			Garveia grisea							
			Lizzia blondina	X	X	X	X	X		
		Halimedusidae	*Tiaricodon coeruleus*	X						not in Med
		Metridiidae	*Metridium senile*					X	X	
		Pandeidae	*Leuckartiara octona*	X	X	X		X		
			Proboscidactyla ornata	X						not in Med
		Porpitidae	*Porpita porpita*				X			
		Tubulariidae	*Ectopleura*				X	X		
			Ectopleura dumortierii	X	X	X				
		Ratkheidae	*Rathkea octopunctata*				X	X		
	Leptothecatae	Aequoreidae	*Aequorea australis*	X			X	X	-	not in Med
			Aequorea macrodactyla	X						
			Aequorea conica	X						
		Blackfordiidae	*Blackfordia virginica*	X			X	X		
		Campanulariidae	*Campanularia subantarctica*	X						not in Med
			Clytia elsaeoswaldae				X			not in Med
			Clytia hemisphaerica				X	X		
			Clytia folleata							
			Clytia gracilis				X	X	+	*Med ?*
			Clytia noliformis				X			
			Clytia sp. GL JRH-2014	X						
			Clytia sp. KC JRH-2012				X			
			Clytia xiamenensis	X						not in Med
			Eucheilota maculata							
			Eucheilota sp. JRH-2014	X						
			Obelia	X			X	X	X	According to 18S this MOTU belongs to either O.dichotoma or O.geniculata, while COI identified it As either O.dichotoma or an undescribed species of Obelia.
		Eirenidae	*Eirene ceylonensis*	X						not in Med
			Eirene brevistylus				X			not in Med
			Eirene pyramidalis	X						not in Med
			Eirene hexamenalis	X						not in Med
			Eutima curva				X	X	+	not in Med

Phylum	Order	Family	0_NCBI TAXONOMY	COI 90%	COI 95%	COI 97%	18S 95%	18S 97%	18S 99%	Notes (WoRMS, WRIMS, Razouls, FISHbase, literature, personal knowledge)
			Eutima gracilis	X	X	X				
			Eutonina indicans							
			Helgicirrha schulzii	X	X	X				
		Plumulariidae	*Plumularia setacea*	X						
		Sugiuridae	*Sugiura chengshanense*	X						not in Med
	Actiniaria	Actiniidae	*Bunodosoma granuliferum*					X	-	not in Med
		Diadunenidae	*Diadumene lineata*	X						
		Sagartiidae	*Sagartiogeton undatus*					X		
	Siphonophora	Clausophyidae	*Chuniphyes multidentata*	X						
		Prayidae	*Gilia reticulata*				X	X		
			Lilyopsis medusa				X	X	+	
		Agalmatidae	*Marrus sp. 1 BO-2009*	X						not in Med
			Nanomia bijuga	X	X	X				
		Apolemidae	*Apolemia sp. 1 YPM 35035*	X						
		Diphyinae	*Muggiaea atlantica*	X	X	X				
			Lensia campanella			X				
	Narcomedusae	Aeginidae	*Solmundella bitentaculata*				X	X	+	not in Med
	Trachymedusae	Geryoniidae	*Liriope tetraphylla*	X			X	X		
		Rhopalonematidae	*Aglantha digitale*				X	X		
			Aglaura hemistoma					X	+	
			Rhopalonema velatum					X	X	
	Rhizostomeae	Rhizostomatidae	*Nemopilema nomurai*	X						
			Rhizostoma pulmo	X	X	X	X	X		
	Limnomedusae	Olindiidae	*Aglauropsis aeora*							
	Alcyonacea	Ellisellidae	*Junceella fragilis*	X			X	X	+	not in Med
Porifera	Bubarida	Dictyonellidae	*Scopalina ruetzleri*	X						
	Clionaida	Spirastrellidae	*Spirastrella*				X	X	X	
	Haplosclerida	Chalinidae	*Haliclona amboinensis*	X						
	Tethyida	Hemiasterellidae	*Axos cliftoni*	X						not in Med
Ctenophora	Beroida	Beroidae	*Beroe*				X	X	+	
	Cydippida		*Mertensia ovum*/Undescribed mertensiid/*Euplokamis sp.*				X	X	X	*Martensia ovum is not present in the Med, while other Martesiids are known to be present here*
		Mertensiidae	Charistephane fugiens	X						
	Lobata	Lampocteridae	*Lampocteis cruentiventer*				X	X	+	not in Med (Pacific)
		Pleurobrachiidae	*Pleurobrachia pileus*					X	+	
Gastrotricha	Macrodasyida	Macrodasyidae	Urodasys sp. 114a	X	X	X				

Table S1. Exhaustive list of taxa recovered at the minimum similarity thresholds of 90%, 95% and 97% for both genes and 99% for 18S only. A plausible misidentification is highlighted in blue, a non-indigenous species (NIS) is in red and a discarded species is in pink. Matches represented by singletons are marked in grey.

	Taxa	Morphology	Metabarcoding	COI	18S
**Cladocera**	*Evadne nordmanni*	x			
	Evadne spinifera	x	x	x	x
	Penilia avirostris	x	x	x	x
	Pleopis polyphemoides	x	x	x	
	Podon intermedius	x	x	x	
	Pseudevadne tergestina	x		x	
**Copepoda**	*Acartia (Acartiura) clausi*	x	x	x	x
	Acartia negligens	x			
	Acartia longiremis	x			
	Calanus finmarchicus		x		x
	Calanus helgolandicus	x	x	x	x
	Calocalanus contractus	x	x	x	
	Calocalanus plumulosus	x			
	Calocalanus styliremis	x			
	Candacia armata		x		x
	Candacia giesbrechti	x			
	Centropages kroyeri	x			
	Clausocalanus arcuicornis	x	x	x	
	Clausocalanus furcatus	x	x	x	
	Clausocalanus jobei	x	x	x	
	Clausocalanus parapergens	x		x	
	Clausocalanus paululus	x	x	x	
	Clausocalanus pergens	x	x	x	
	Ctenocalanus vanus	x	x	x	
	Copilia quadrata	x			
	Diaixis hibernica		x	x	
	Diaixis pygmaea	x			
	Ditrichocorycaeus brehmi	x			
	Euterpina acutifrons	x	x	x	x
	Farranula	x			
	Goniopsyllus rostratus	x	x	x	
	Haloptilus longicornis	x			
	Isias clavipes	x			x
	Mecynocera clausi	x			
	Mesocalanus tenuicornis	x	x	x	x
	Microcalanus		x	x	
	Microsetella norvegica	x			
	Microsetella rosea	x			
	Nannocalanus minor	x	x	x	
	Neocalanus gracilis	x			
	Oithona nana	x	x		x
	Oithona plumifera	x			
	Oithona setigera	x			
	Oithona similis	x	x	x	x
	Oncaea curta	x			
	Oncaea media	x			
	Oncaea mediterranea	x	x	x	
	Oncaea scottodicarloi		x	x	
	Oncaea venusta	x	x	x	x
	Oncaea waldemari		x	x	

Taxa	Morphology	Metabarcoding	COI	18S
Paracalanus denudatus	x			
Paracalanus nanus	x		x	
Paracalanus indicus		x	x	
Paracalanus parvus	x	x	x	x
Paracalanus quasimodo		x	x	
Paraeuchaeta hebes	x	x	x	
Pleuromamma gracilis	x	x	x	
Pleuromamma robusta		x		x
Pseudocalanus elongatus		x	x	x
Pseudocalanus moultoni		x		x
Pseudodiaptomus marinus	x	x	x	x
Sapphirina	x			
Temora longicornis	x	x	x	
Temora stylifera	x	x	x	
Triconia dentipes	x			
Triconia umerus		x	x	
Tritia		x	x	

Table S2. List of taxa identified by morphological and/or DNA metabarcoding approaches (COI and 18S).

Station	cruise	Date	time	Latitude N	Longitude E	Depth
4	Echosurvey	8/27/2014	09:57	43°48.43	13°09.75	13.0
6	Echosurvey	8/27/2014	13:47	44°00.28	13°23.92	61.4
8	Echosurvey	8/27/2014	15:58	44°12.56	13°38.67	63.8
12	Echosurvey	8/29/2014	23:13	44°29.993	13°08.026	41.4
14	Echosurvey	8/30/2014	01:48	44°18.81	12°52.90	41.8
16	Echosurvey	8/30/2014	04:12	44°07.47	12°37.54	11.0
20	Echosurvey	8/30/2014	17:03	44°30.14	12°23.87	13.0
22	Echosurvey	8/30/2014	20:53	44°39.14	12°45.49	34.6
24	Echosurvey	8/30/2014	23:14	44°47.34	13°05.83	40.0
25	Echosurvey	8/31/2014	02:30	44°42.526	12°26.790	21.0
26	Echosurvey	8/31/2014	04:41	44°55.236	12°35.155	16.3
28	Echosurvey	8/31/2014	08:05	44°58.47	13°04.76	35.0
30	Echosurvey	9/1/2014	12:42	45°40.00	13°32.88	16.0
32	Echosurvey	9/1/2014	15:26	45°34.41	13°42.05	18.0
33	Echosurvey	9/1/2014	17:11	45°33.01	13°34.33	22.0
35	Echosurvey	9/1/2014	21:43	45°43.06	13°39.52	16.5
36	Echosurvey	9/2/2014	00:25	45°39.24	13°13.65	14.3
37	Echosurvey	9/2/2014	01:26	45°32.77	13°18.58	20.7
38	Echosurvey	9/2/2014	04:31	45°31.306	12°50.636	17.0
40	Echosurvey	9/4/2014	17:17	45°22.96	13°07.50	28.6
41	Echosurvey	9/4/2014	18:19	45°15.84	13°03.02	31.0
42	Echosurvey	9/4/2014	20:35	45°27.82	12°41.73	19.0
43	Echosurvey	9/4/2014	21:36	45°22.99	12°32.77	19.0
44	Echosurvey	9/5/2014	06:46	45°16.65	12°46.75	27.0
45	Echosurvey	9/5/2014	10:04	45°15.12	12°24.53	21.0
46	Echosurvey	9/5/2014	11:24	45°05.30	12°26.24	22.6
48	Echosurvey	9/5/2014	14:29	45°09.41	12°43.63	30.6
50	Echosurvey	9/5/2014	16:22	45°07.46	13°02.13	34.3
52	Echosurvey	9/7/2014	14:46	43°38.79	13°35.79	19.8
54	Echosurvey	9/7/2014	16:47	43°46.51	13°53.58	74.6
56	Echosurvey	9/7/2014	18:46	43°53.72	14°10.42	72.4
58	Echosurvey	9/8/2014	07:52	43°27.99	14°32.92	88.8
60	Echosurvey	9/8/2014	11:25	43°19.97	14°13.29	75.3
62	Echosurvey	9/8/2014	13:49	43°11.37	13°52.36	14.5
65	Echosurvey	9/9/2014	04:20	42°41.47	14°04.70	14.8
66	Echosurvey	9/9/2014	08:44	42°45.72	14°18.42	87.6
67	Echosurvey	9/9/2014	10:28	42°49.88	14°32.25	206.4
70	Echosurvey	9/9/2014	23:24	42°39.26	15°18.26	135.0
73	Echosurvey	9/10/2014	02:26	42°28.74	14°56.62	154.6
76	Echosurvey	9/10/2014	06:45	42°18.09	14°34.39	22.3
79	Echosurvey	9/10/2014	21:41	41°58.89	15°06.13	17.1
82	Echosurvey	9/11/2014	00:53	42°13.66	15°31.24	115.0
85	Echosurvey	9/11/2014	04:06	42°27.61	15°55.25	129.7
89	Echosurvey	9/13/2014	14:12	41°58.79	16°02.99	21.0
90	Echosurvey	9/13/2014	16:30	42°09.73	16°17.13	127.5
94	Echosurvey	9/16/2014	12:03	42°41.50	14°04.68	15.3

Table S3. Details of the position of the stations, date of sampling and respective depths in metres.
